# A novel network regularized matrix decomposition method to detect mutated cancer genes in tumour samples with inter-patient heterogeneity

**DOI:** 10.1038/s41598-017-03141-w

**Published:** 2017-06-06

**Authors:** Jianing Xi, Ao Li, Minghui Wang

**Affiliations:** 1 School of Information Science and Technology, University of Science and Technology of China, Hefei, AH230027 China; 2 Centers for Biomedical Engineering, University of Science and Technology of China, Hefei, AH230027 China

## Abstract

Inter-patient heterogeneity is a major challenge for mutated cancer genes detection which is crucial to advance cancer diagnostics and therapeutics. To detect mutated cancer genes in heterogeneous tumour samples, a prominent strategy is to determine whether the genes are recurrently mutated in their interaction network context. However, recent studies show that some cancer genes in different perturbed pathways are mutated in different subsets of samples. Subsequently, these genes may not display significant mutational recurrence and thus remain undiscovered even in consideration of network information. We develop a novel method called mCGfinder to efficiently detect mutated cancer genes in tumour samples with inter-patient heterogeneity. Based on matrix decomposition framework incorporated with gene interaction network information, mCGfinder can successfully measure the significance of mutational recurrence of genes in a subset of samples. When applying mCGfinder on TCGA somatic mutation datasets of five types of cancers, we find that the genes detected by mCGfinder are significantly enriched for known cancer genes, and yield substantially smaller p-values than other existing methods. All the results demonstrate that mCGfinder is an efficient method in detecting mutated cancer genes.

## Introduction

Next generation sequencing (NGS) technology has revolutionized the detection of somatic mutations in cancer genomics in recent years^1–5^. With NGS technique, large numbers of tumour samples have been sequenced in projects such as The Cancer Genome Atlas (TCGA)^6^ and the International Cancer Genome Consortium (ICGC)^7–10^. These projects provide excellent opportunities to find mutated cancer genes from a large cohort of tumour samples, which can help differentiate functionally related driver mutations from passenger mutations^11–13^. A common strategy to detect mutated cancer genes is to detect genes with significant mutational recurrence^14, 15^. Although some cancer genes show high mutation frequencies (such as TP53 or KRAS, both well-known cancer genes), previous studies demonstrate that extensive inter-patient heterogeneity is present in various types of cancers^9^ and some cancer genes are mutated in a small number of samples^16–18^. These mutated cancer genes are not likely to display significant mutational recurrence due to inter-patient heterogeneity, and consequently they may be underestimated by the frequency-based methods^13, 17–19^.

A prominent explanation of inter-patient heterogeneity is that the behavior of key pathways of tumour samples is perturbed by mutated cancer genes, and only a subset of genes in these pathways are mutated in a given sample^17–19^. Subsequently, many recent approaches exploit large scale gene interaction network as an additional source to identify cancer genes mutated in perturbed pathways^17–21^. Considering both mutation frequencies of genes and information from interaction network such as iRefIndex^22^, HPRD^23^, STRING^24^ and others^25–27^, these approaches detect mutated cancer genes by determining whether the investigated genes are recurrently mutated in their network context. For example, HotNet^17, 18^ and HotNet2^19^ propagate the “heat” of mutation frequencies of genes through the network and select genes with significantly high “heat” scores as mutated cancer genes. ReMIC^20^ detects genes with mutational recurrence in their network context through a diffusion graph kernel strategy. In these network-based approaches, mutated cancer genes are determined according to both their mutational recurrence and the mutational influence from their network context.

Despite the success achieved by the aforementioned approaches, another important aspect contributing to inter-patient heterogeneity is that some cancer genes in different perturbed pathways are mutated in different subsets of samples, which has been observed in recent studies^28–31^. For example, transcriptional abnormalities of some genes in different pathways are found in different subsets of samples^28–30^. Moreover, another study shows that in multiple types of cancers, somatic mutations of some cancer genes in various perturbed subnetworks are observed in distinct subgroups, suggesting that cancer genes in different pathways may be mutated in different subsets of samples^31^. If some mutated cancer genes are associated with only a subset of samples, these genes may not exhibit significant mutational recurrence in all samples even in consideration of the mutational influence from their network context. Accordingly, these cancer genes are likely to be underestimated by the existing methods, as these methods are not specially designed for cancer gene detection under this scenario.

Identifying abnormal genes in a subset of samples from cancer data with inter-patient heterogeneity is a critical problem in bioinformatics, and therefore has been studied in many previous researches^32–35^. To tackle this problem, methods based on matrix decomposition framework have been introduced^36–40^. These methods decompose the cancer data matrix into different components, which indicate different subsets of samples and the related abnormal genes. Nevertheless, to the best of our knowledge, these matrix decomposition based methods cannot efficiently incorporate information from network context. Therefore, to capture the mutated cancer genes in perturbed pathways associated with only a subset of samples, it is an urgent need to establish an integrated method that can both incorporate gene interaction network information and measure the significance of mutational recurrence of genes in a subset of samples.

In this study, we propose a novel method called mCGfinder, to detect mutated cancer genes in tumour samples with inter-patient heterogeneity. Based on matrix decomposition framework, mCGfinder can successfully measure the significance of mutational recurrence of genes in a subset of samples instead of in all samples. Meanwhile, we introduce graph Laplacian regularization^41^ into mCGfinder, which can efficiently measure the mutational influence from the network neighbors of the investigated genes. When applying mCGfinder on TCGA somatic mutation datasets of five types of cancers, we find that the genes detected by mCGfinder are significantly enriched for known cancer genes. Notably, mCGfinder yields substantially smaller p-values (e.g., p-value = 1.24e–17 for breast cancer) than other existing network-based approaches across all investigated cancers. Moreover, we observe that high percentages of known cancer genes are included in the top ranked genes detected by mCGfinder. All the results indicate the efficiency of mCGfinder in detecting mutated cancer genes in heterogeneous tumour samples.

## Results

### Overview of mCGfinder

**Figure 1:**
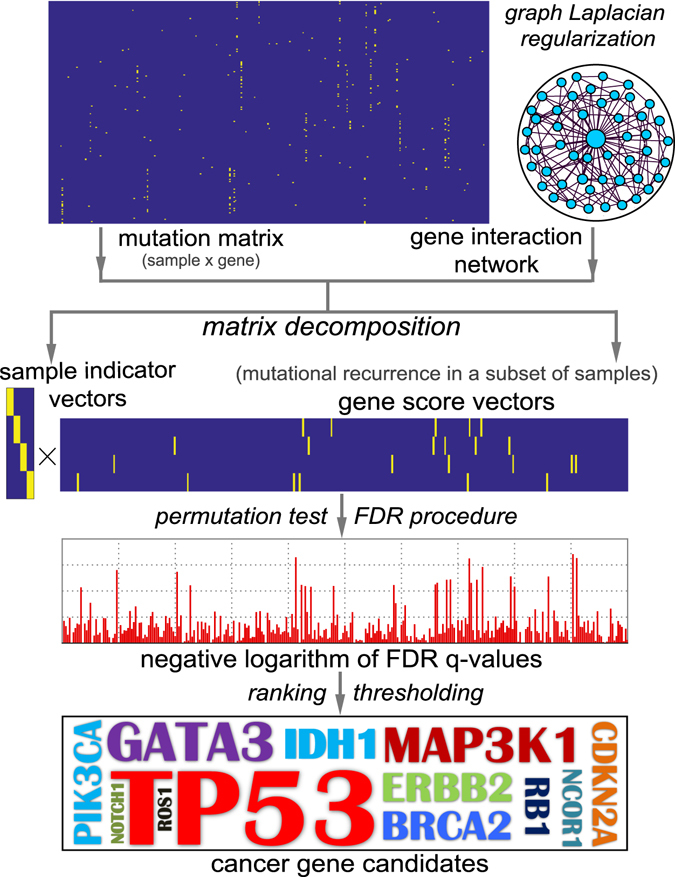
Schematic diagram of mCGfinder, which is a matrix decomposition method integrated with network information. It decomposes the mutation matrix as the matrix multiplication of the sample indicator vectors and the transpose of gene score vectors. The different components in the results of mCGfinder are regarded as the outer products of different sample indicator vectors and their related gene score vectors, where the summation of the components is an approximation of the mutation matrix. Graph Laplacian regularization is used to incorporate information of gene interaction network into mCGfinder. After the matrix decomposition procedure, the mutational recurrence of genes in different subsets of samples can be measured from the gene score vectors of the related component, and the related subsets samples of the component are indicated by the sample indicator vectors. Through permutation test and false discovery rate (FDR) control, mutated cancer gene candidates can be identified by thresholding FDR q-values of the genes.

To detect mutated cancer genes from somatic mutation data of inter-patient heterogeneous cancers, mCGfinder involves mainly three steps (Fig. 1). In the first step, mCGfinder decomposes the mutation data matrix of heterogeneous tumour samples into several components, and use the summation of these components to approximate the mutation matrix. Each component obtained by mCGfinder is the outer product of sample indicator vector and gene score vector, indicating a subset of samples and the mutational recurrence of genes related to these samples respectively. At the same time, we also use graph Laplacian regularization to incorporate information of gene interaction network into mCGfinder. In the second step, we apply permutation test and false discovery rate (FDR) control on gene score vectors of every components, and obtain the FDR q-values of all investigated genes. In the third step, mutated cancer genes are selected with FDR q-values less than the default significance threshold 0.05^29, 42^. The code of mCGfinder can be freely accessed at https://github.com/USTC-HIlab/mCGfinder.

### Comparison analysis

**Figure 2:**
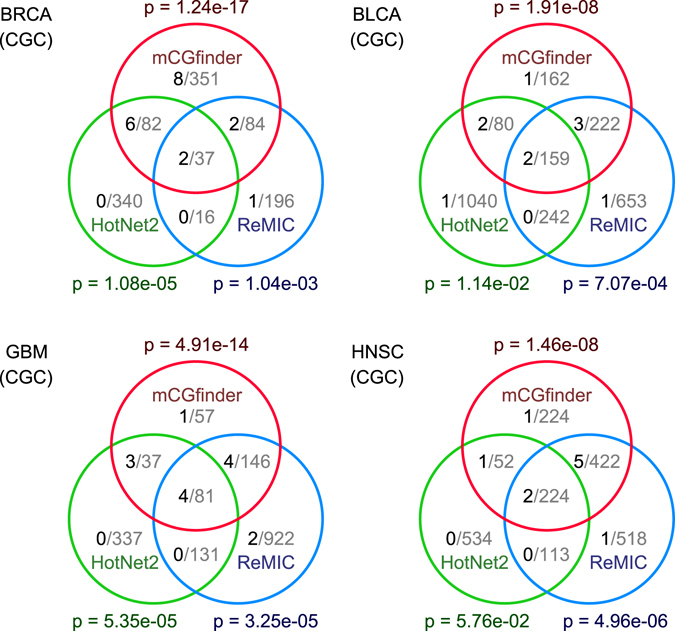
Venn diagrams of intersections between the genes detected by mCGfinder (red circle), HotNet2 (green circle) and ReMIC (blue circle) on TCGA somatic mutation datasets of BRCA (north-west panel), BLCA (north-east panel), GBM (south-west panel) and HNSC (south-east panel). The gray and black numbers in each region of the Venn diagrams indicate the number of detected genes and the number of genes also reported in Cancer Gene Census (CGC)^46^ respectively. The p-values next to the circles of the methods are calculated by Fisher’s exact test, representing the enrichment significance of the detection results for CGC annotated cancer genes.

For the analysis of mCGfinder in mutated cancer gene detection, we employ TCGA somatic mutation data of five types of cancers in this study, including 776 breast invasive carcinoma (BRCA) samples^29^, 238 bladder urothelial carcinoma (BLCA) samples^30^, 291 glioblastoma multiforme (GBM) samples^43^, 509 head and neck squamous cell carcinoma (HNSC) samples^44^ and 197 acute myeloid leukemia (LAML) samples^45^. The performance of mCGfinder is compared against two existing methods, HotNet2^19^ and ReMIC^20^. In mCGfinder, HotNet2 and ReMIC, we use a highly curated gene interaction network iRefIndex^22^ as the network information. In the comparison study, mCGfinder, HotNet2 and ReMIC are configured by their default settings^19, 20^ (details in Supplementary materials). An overview of the mutated cancer genes detected by mCGfinder, HotNet2 and ReMIC is illustrated as Venn diagrams (Fig. 2 and Supplementary Fig. S5A). For all the five types of cancers, there is a high concordance between the results of mCGfinder and the results of the other two methods. Among the genes detected by mCGfinder, the percentages of genes that are also detected by at least one of the other methods range from 36.6% (BRCA) to 84.3% (LAML) across the five types of cancers.

In this study, we apply Fisher’s exact test on the detection results to evaluate whether the detected genes of the three methods are significantly enriched for known mutated cancer genes in Cancer Gene Census (CGC)^46^, which is a highly curated database of cancer genes. For all the five types of cancers, the results of mCGfinder are highly enriched for CGC cancer genes (Fig. 2), and yield the most significant p-values among the three investigated methods. Taking BRCA as an example, HotNet2 and ReMIC obtain p-values of 1.08e-05 (8 CGC genes) and 1.04e-03 (5 CGC genes) respectively, which suggest that these results are significantly different than random selection. In comparison, mCGfinder achieves a p-value of 1.24e-17 and captures 18 CGC breast cancer genes. Notably, there are 8 CGC genes (AKT1, BRCA2, CASP8, CTCF, MAP3K1, MAP3K13, NCOR1 and TBX3) captured by mCGfinder but not by HotNet2 or ReMIC. Literature survey shows that AKT1 gene is implicated as significantly mutated gene in breast cancer in a previous study^29^, and mutations of BRCA2 gene are reported to be involved in the primary events of breast carcinogenesis^47^. In the three other types of cancers, mCGfinder also provides high enrichment for CGC genes, with associated p-values of 1.91e-08 in BLCA (8 CGC genes), 4.91e-14 in GBM (12 CGC genes), 1.46e-08 in HNSC (9 CGC genes) and 5.57e-16 in LAML (10 CGC genes). Interestingly, for all the investigated cancers, the genes detected by both HotNet2 and ReMIC but not by mCGfinder include no known CGC gene (Fig. 2). Taking BLCA as an example, there is no CGC gene among the 242 genes detected by both HotNet2 and ReMIC but not by mCGfinder. The full lists of CGC genes detected by mCGfinder on the five types of cancers are provided in Supplementary Table S1.

To give a more comprehensive assessment on the detection results, we also use another independent curated cancer gene database, Integrative Onco Genomics (IntOGen)^48^. In the enrichment analysis for known cancer genes reported in IntOGen, mCGfinder demonstrates comparable or better performance than the two competing methods (Supplementary Figs S1 and S5A). In BRCA, the detection results of HotNet2 and ReMIC show good performance and contain 22 and 31 IntOGen breast cancer genes respectively. In comparison, mCGfinder successfully recovers 60 IntOGen breast cancer genes. The enrichment p-values of the results of mCGfinder, HotNet2 and ReMIC for IntOGen genes in BRCA are 1.97e-36, 1.74e-06 and 1.15e-16 respectively. For BLCA data, there are 31 and 44 IntOGen genes captured by HotNet2 (p-value = 4.16e-03) and ReMIC (p-value = 1.30e-10) respectively. In comparison, mCGfinder predicts 56 IntOGen genes, yielding a p-value of 4.78e-34. The IntOGen genes detected by mCGfinder on the five types of cancers are listed in Supplementary Table S2. Finally, we perform cancer gene enrichment analysis by using the combined cancer gene lists of both CGC and IntOGen databases, and similar conclusion can be drawn from the results across all the five types of cancers (Supplementary Figs S1 and S5A). The CGC and IntOGen genes identified by mCGfinder but not by the other investigated methods along with their functions are demonstrated in Supplementary Table S3.

### Ranking analysis

**Figure 3:**
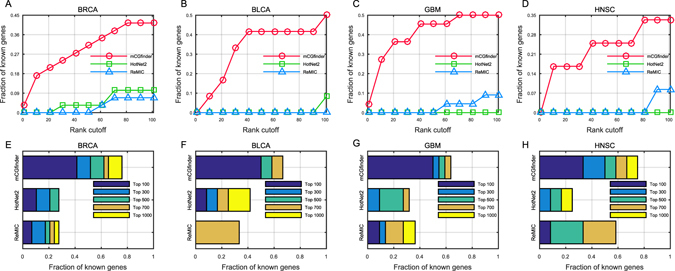
Rank cutoff curves of top 100 candidates in mCGfinder (red line with circle markers), HotNet2 (green line with square markers) and ReMIC (blue line with triangle markers) results, describing the relation between various cutoffs and the fraction of known CGC cancer genes ranked above this cutoff in BRCA (**A**), BLCA (**B**), GBM (**C**) and HNSC (**D**). Cumulative fractions of known CGC cancer genes annotated by CGC within the top 100, 300, 500, 700 and 1000 genes in BRCA (**E**), BLCA (**F**), GBM (**G**) and HNSC (**H**). Results from all the assessments indicate the generally improved performance of mCGfinder over the competing methods.

In addition to the statistical enrichment analysis, in order to comprehensively evaluate the performances of mCGfinder, we further use the results obtained by not only the default threshold but also various thresholds by following previous studies^49–52^. The gene ranking scores of different approaches are detailed in Supplementary materials. By raising the threshold and obtaining the percentages of known cancer genes falling under the category, we can evaluate the detection results of different methods comprehensively through rank cutoff curves^49, 50^. Here we use the rank cutoff curves as the evaluation metric for the top ranked genes detected by the investigated methods, which are drawn by listing the percentages of known cancer genes that are also included in the top ranked genes. As shown in Fig. 3A–D, the top ranked genes detected by mCGfinder contain consistently higher percentages of known CGC genes than the results of the other methods at various rank thresholds. Taking BRCA as an example, 3.5% of CGC breast cancer genes are included in the top 50 genes detected by HotNet2. In comparison, the top 50 genes identified by mCGfinder contain 31.0% of CGC breast cancer genes. When the rank threshold of genes raises to 100, the percentages of known cancer genes detected by HotNet2 and ReMIC also increase to 10.3% and 6.9% respectively. In comparison, there are 41.4% of known CGC genes included in the results of mCGfinder. Similarly, in the other types of cancers, the top ranked genes detected by mCGfinder also contain high fractions of known cancer genes. For example, there are 50.0%, 50.0% and 33.3% of known CGC genes included in the top 100 genes detected by mCGfinder on BLCA, GBM and HNSC data respectively.

Next, we assess the performance of different methods with larger rank thresholds, and mCGfinder still compares favorably to the competing methods across all the five types of cancers (Fig. 3E–H and Supplementary Fig. S5C). When the rank threshold raises to 500, both HotNet2 and ReMIC demonstrate reasonable performance in BRCA and yield percentages 27.6% and 20.7% of CGC cancer genes respectively. In comparison, mCGfinder achieves a percentage of 62.7%. In BLCA, GBM and HNSC, more than half of the known CGC cancer genes are also included in the top 500 genes detected by mCGfinder respectively. We further assess the top ranked genes of the investigated methods by IntOGen gene lists and the combined gene lists of both the two databases. The results also show that mCGfinder achieves the highest percentages among the three investigated methods throughout the rank cutoff analysis (Supplementary Figs S2, S3 and S5B,C).

**Figure 4:**
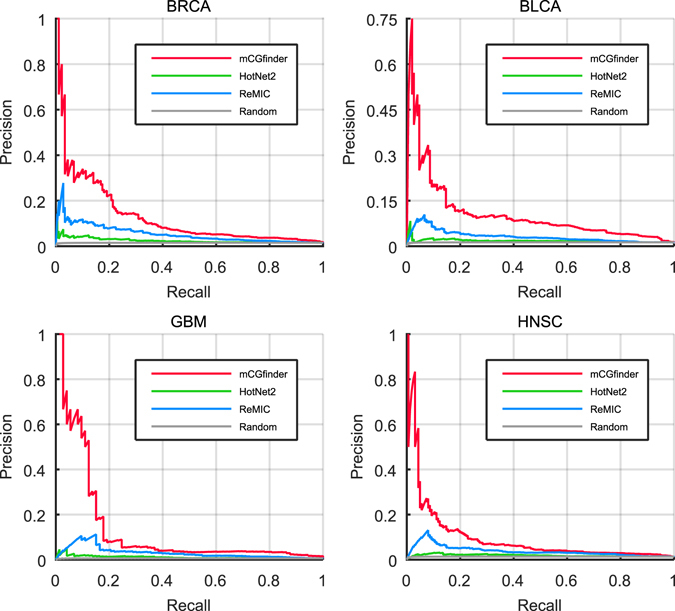
Precision-recall curves for the three methods on BRCA (north-west panel), BLCA (north-east panel), GBM (south-west panel) and HNSC (south-east panel) data, where red, green, blue and gray lines represent the curves of mCGfinder, HotNet2, ReMIC and random selection respectively. For each curve, the points indicate the precisions and recalls at different ranks in the prediction results. The precision is computed as the fraction of the top ranked genes that are known cancer genes, and the recall is computed as the fraction of known cancer genes in the top ranked genes.

Moreover, by varying the rank thresholds and calculating the precisions and recalls, we draw the precision-recall curve (PR curve) of the results detected by the investigated methods as the assessment metric used in previous studies^51, 52^. For all the five types of cancers, when the known cancer genes in CGC are used as gold-standard, the PR curves of mCGfinder are clearly located over the curves of the other methods (Supplementary Figs S4 and S5D). As the limited number of known cancer genes from CGC may lead to inaccurate performance, we further use known cancer genes annotated by IntOGen for evaluation (Fig. 4 and Supplementary Fig. S5D), in which the number of known breast cancer genes is largely increased. Taking BRCA as an example, when the recalls are fixed at 10.0%, the precisions of mCGfinder, HotNet2 and ReMIC are 33.9%, 4.3% and 11.9% respectively, which are consistently better than random selection. The area under the precision-recall curve of mCGfinder is also greater than the other methods (Supplementary Table S4). In consistent with BRCA results, mCGfinder also gives the best performance among the detection results of the investigated methods on BLCA, GBM, HNSC and LAML data when evaluated by IntOGen. Similar conclusions can also be obtained from analysis of the detection results from the combined gene lists of the two databases (Supplementary Figs S4, S5D and Supplementary Table S4).

### Computational cost

**Figure 5:**
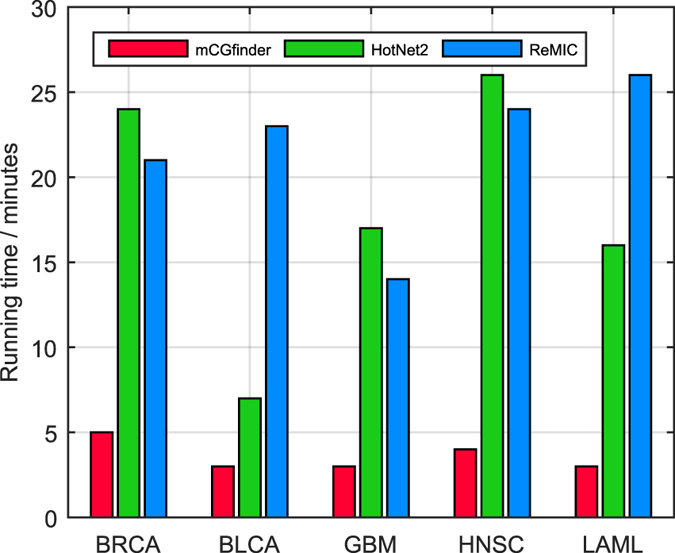
Running time comparison of mCGfinder (red bar), HotNet2 (green bar) and ReMIC (blue bar) on datasets of BRCA, BLCA, GBM, HNSC and LAML respectively.

In addition to the analysis of detection performance, we further examine the computational time of the three investigated methods. The experiments in this study are performed on a computer with Intel Xeon(R) CPU E5-2630 0 @ 2.30 GHz × 18 Processors and 64 GB of memory. For BRCA, BLCA, GBM, HNSC and LAML somatic mutation datasets with 12129 genes and 776, 238, 291, 509 and 197 samples, the running time of mCGfinder is 3–5 minutes in average, which is smaller than HotNet2 and ReMIC (Fig. 5). For example, in BRCA, HotNet2 takes around 24 minutes, and ReMIC takes around 21 minutes. In comparison, mCGfinder takes only around 5 minutes. In HNSC, mCGfinder, HotNet2 and ReMIC take around 4, 24 and 26 minutes respectively.

## Discussion

Developing efficient methods to detect cancer genes from inter-patient heterogeneous tumour samples is an challenging task, and a major obstacle is the fact that some cancer genes are mutated in perturbed pathways associated with only a subset of samples^28, 31^. Thus, these mutated cancer genes may not be significantly recurrent in all samples and remain undiscovered even when the mutations in their interaction network context are considered. In this paper, based on the combination of matrix decomposition framework and information from gene interaction network, we propose a novel method which is capable of detecting mutated cancer genes in a subset of samples. When applied on TCGA somatic mutation datasets of five types of cancers, mCGfinder precisely recovers many known cancer genes. Our results also show that the performance of mCGfinder is not sensitive to the selection of the tuning parameter (Supplementary Fig. S6). Notably, mCGfinder achieves the highest enrichment for known genes among the investigated methods, suggesting that it is a powerful bioinformatics tool for mutated cancer gene detection.

A significant distinction between mCGfinder and the existing network-based approaches for cancer gene detection is that mCGfinder decomposes the mutation data matrix of heterogeneous tumour samples into different components, and measures the mutational recurrence of genes in subsets of samples indicated by the components. Based on this design, mCGfinder greatly complements the detection results of the existing approaches. Nevertheless, it should be pointed out that the evaluation results are not sufficient to mean as a criticism of the other investigated methods. Instead, they show the difference between whether considering the mutated cancer genes in different subsets of samples or not. In the detection results of the investigated cancers, some CGC genes missed by mCGfinder are detected either by HotNet2 only or by ReMIC only (Fig. 2). For example, in BLCA results there is 1 CGC gene among the 1040 genes detected by HotNet2 only, and there is 1 CGC gene among the 653 genes detected by ReMIC only. These results suggest that it may be worth using both mCGfinder and the existing methods to maximize the detection rate of mutated cancer genes.

Despite the promising results achieved by the purposed method, there are also some avenues for further investigation. For example, our method is not designed to address the issue of intra-tumour heterogeneity^9^, which cannot be represented by the input binary matrix. In consistent with HotNet2 and ReMIC which highly depend on gene interaction network, our method utilizes gene interaction network as an important information source for detecting mutated cancer genes. Therefore, mCGfinder is not yet applicable for genes that are not included in gene interaction network. Also, it is noteworthy that unlike previous approach^31^, our method is not designed to stratify cancer samples and cannot incorporate biological knowledge of cancer subtypes^29^. Meanwhile, the objective function in mCGfinder is not guaranteed to be convex albeit a local optimum can be reached. Furthermore, a promising expansion to the mCGfinder in future work would be to integrate information from not only gene interactions, but also different types of information such as copy number alternation, gene expression and DNA methylation, which would offer an opportunity to comprehensively understand cancer events from a multi-omics view^51, 53, 54^.

In summary, mCGfinder is a novel method to efficiently detect mutated cancer genes in tumour samples with inter-patient heterogeneity, which provides a more sophisticated view of cancer genomics from both the influence of interaction network context and mutational recurrence of genes in different subsets of samples. Altogether, mutational profile analysis from mCGfinder and further experimental follow-up may help take a step forward to a more comprehensive knowledge of the cancer genome.

## Materials and Methods

### TCGA somatic mutation data of cancers

We apply mCGfinder on TCGA somatic mutation data of five types of cancers, BRCA, BLCA, GBM, HNSC and LAML (detailed information in Supplementary materials and Supplementary Table S5). For each type of cancer, the mutation data is a binary matrix **X** = (*X*_*ij*_)_*n*×*p*_ where the rows and columns of the mutation matrix denote the tumour samples (totally *n* samples) and the investigated genes (totally *p* genes) respectively. Each entry *X*_*ij*_ of the matrix indicates the binary state of the gene, in which 1 represents the *i*-th sample contains a somatic mutation of the *j*-th gene, and 0 otherwise^31, 55^.

### Network regularized matrix decomposition

Based on matrix decomposition framework, mCGfinder decomposes the matrix **X** of somatic mutation data in heterogeneous tumour samples into different components, and the summation of these components can be regarded as an approximation of the mutation data matrix, i.e.

1 $$\begin{array}{l}{\boldsymbol{X}}=\sum _{r=1}^{R}{{\boldsymbol{s}}}_{r}{{\boldsymbol{g}}}_{r}^{{\rm{T}}}+{{\boldsymbol{\varepsilon }}}_{r}\mathrm{.}\end{array}$$

where ${{\boldsymbol{s}}}_{r}={({s}_{ir})}_{n\times 1}$ and ${{\boldsymbol{g}}}_{r}={({g}_{jr})}_{p\times 1}$ are the sample indicator vector and the gene score vector for the *r*-th component. The **ε**_*r*_ is the residual matrix for the *r*-th component, and *R* is the total number of the components obtained by mCGfinder. The sample indicator vector ***s***_*r*_ indicates the assignment of tumour samples to the *r*-th component, in which the coefficient *s*_*ir*_ = 1 represents that the *i*-th samples are included in the component, and *s*_*ir*_ = 0 otherwise. As for the gene score vector ***g***_*r*_ of the *r*-th component, a higher value of the coefficient *g*_*jr*_ of the vector presents a larger potential of the *j*-th gene to be a mutated cancer gene. Note that the first component, which is the outer product of the two vectors ${{\boldsymbol{s}}}_{1}{{\boldsymbol{g}}}_{1}^{{\rm{T}}}=({s}_{i1}{g}_{j1}{)}_{n\times p}$, is the best rank-one approximation of the data matrix ***X***. Thus, we can use the approximation to decompose the first component (***S***_1_ and ***g***_1_) from the data matrix, and obtain the remaining components through a component-by-component strategy^36, 37, 40^. Also, to efficiently incorporate information from gene interaction network, we use graph Laplacian regularization on the gene score vector ***g***_1_. Subsequently, we construct an optimization problem for vector ***s***_1_ and ***g***_1_ to obtain the first component, and the objective function is,

2 $$\begin{array}{c}\mathop{{\rm{m}}{\rm{i}}{\rm{n}}}\limits_{{{\boldsymbol{s}}}_{1},{{\boldsymbol{g}}}_{1}}{||{\boldsymbol{X}}-{{\boldsymbol{s}}}_{1}{{\boldsymbol{g}}}_{1}^{{\rm{T}}}||}_{F}^{2}+{\lambda }_{L}{{\boldsymbol{g}}}_{1}^{{\rm{T}}}{\boldsymbol{L}}{{\boldsymbol{g}}}_{1}\\ {\rm{s}}{\rm{.t}}{\rm{.}}{{s}}_{1}\in {\mathrm{\{0,1\}}}^{n}\mathrm{.}\end{array}$$

where ${||\cdot ||}_{F}^{2}$ denotes the squared Frobenius norm of a matrix, and ${{\boldsymbol{s}}}_{1}\in {\mathrm{\{0,1\}}}^{n}$ indicates that the coefficients in vector **s**_1_ can be either 1 or 0. The matrix ${\boldsymbol{L}}={({L}_{ij})}_{p\times p}$ is the Laplacian matrix of the gene interaction network, which is calculated through the matrix subtraction ${({L}_{ij})}_{p\times p}={({D}_{ij})}_{p\times p}-{({A}_{ij})}_{p\times p}$. The matrix ${({A}_{ij})}_{p\times p}$ is the symmetric normalized adjacency matrix of the gene interaction network (see Supplementary materials for details of the normalization procedure), and the matrix ${({D}_{ij})}_{p\times p}$ is a diagonal matrix whose entries are the column sums of matrix ${({A}_{ij})}_{p\times p}$.

In the objective function (2), the first term is the summation of the residuals between the first component and the data matrix. When the first term is minimized, we can obtain a component that best fit the data matrix. The second term is the graph Laplacian term, which can be rewritten as

3 $$\begin{array}{l}{{\boldsymbol{g}}}_{1}^{{\rm{T}}}{\boldsymbol{L}}{{\boldsymbol{g}}}_{1}=\sum _{i\mathrm{=1}}^{p}\sum _{j\mathrm{=1}}^{p}{g}_{i1}{g}_{j1}{L}_{ij}=\frac{1}{2}\sum _{i\mathrm{=1}}^{p}\sum _{j=i+1}^{p}{({g}_{i1}-{g}_{j1})}^{2}{A}_{ij}\mathrm{.}\end{array}$$

Through the graph Laplacian term, we can successfully adopt the assumption that if the *i*-th gene and the *j*-th gene are connected in the gene interaction network (*A*_*ij*_ > 0), the scores *g*_*i*1_ and *g*_*j*1_ of the two genes are also close to each other. The tuning parameter *λ*_*L*_ is used to balance the fitness of the model (first term) and the smoothness of the scores of connected genes (second term), which is set to 0.1 in this study. Accordingly, mCGfinder can efficiently measure the significance of mutational recurrence of genes in a subset of samples and incorporate information from network context at the same time.

### Iterative estimation procedure

To solve the optimization problem in (2), we employ an efficient iterative procedure to estimate the two vector ***s***_1_ and ***g***_1_ alternatively^36, 37, 40^. When the gene score vector ***g***_1_ is fixed, the optimization function to solve the coefficient *s*_*i*1_ in the sample indicator vector ***s***_1_ is formulated as below:

4 $$\begin{array}{c}\mathop{{\rm{m}}{\rm{i}}{\rm{n}}}\limits_{{s}_{i1}}\,{s}_{i1}^{2}{||{{\boldsymbol{g}}}_{1}||}_{2}^{2}-{s}_{i1}{\mathrm{(2}{\boldsymbol{X}}{{\boldsymbol{g}}}_{1})}_{i}\\ {\rm{s}}{\rm{.t}}{\rm{.}}\,{s}_{i1}({s}_{i1}-\mathrm{1)}=\mathrm{0,}\,\forall i=\mathrm{1,}\ldots ,n,\end{array}$$

where the ${\Vert \cdot \Vert }_{2}^{2}$ denotes the squared L2-norm of a vector, and (·)_*i*_ indicates the *i*-th coefficients of a vector. Since the values of the coefficients in sample indicator vector are constrained to be binary, we introduce Boolean constraint on coefficients in vector ***s***_1_^56^. For the assignment of the *i*-th sample of the first component, the estimation of *s*_*i*1_ in vector ***s***_1_ can be calculated through Karush-Kuhn-Tucker (KKT) conditions,

5 $$\begin{array}{l}{s}_{i1}=(\begin{array}{ll}1 & {\rm{if}}\,{\mathrm{(2}{\boldsymbol{X}}{{\boldsymbol{g}}}_{1})}_{i}\ge {||{{\boldsymbol{g}}}_{1}||}_{2}^{2}\\ 0 & {\rm{otherwise}}\mathrm{.}\end{array}\end{array}$$

Likewise, when the sample indicator vector ***s***_1_ is fixed, the optimization function to solve the gene score vector ***g***_1_ in optimization problem (2) is formulated as below:

6 $$\mathop{{\rm{m}}{\rm{i}}{\rm{n}}}\limits_{{{\bf{g}}}_{1}}\,({\Vert {{\boldsymbol{s}}}_{1}\Vert }_{2}^{2}){{\boldsymbol{g}}}_{1}^{{\rm{T}}}{{\boldsymbol{g}}}_{1}-2({{\boldsymbol{X}}}^{{\rm{T}}}{{\boldsymbol{s}}}_{1}){{\boldsymbol{g}}}_{1}+{\lambda }_{L}{{\boldsymbol{g}}}_{1}^{{\rm{T}}}{\boldsymbol{L}}{{\boldsymbol{g}}}_{1}\mathrm{.}$$

Similar to the derivation for the sample indicator vector above, the gene score vector can also be obtained through the KKT conditions:

7 $$\begin{array}{l}{{\boldsymbol{g}}}_{1}={({\Vert {{\boldsymbol{s}}}_{1}\Vert }_{2}^{2}{{\boldsymbol{I}}}_{p}+{\lambda }_{L}{\boldsymbol{L}})}^{-1}({{\boldsymbol{X}}}^{{\rm{T}}}{{\boldsymbol{s}}}_{1}),\end{array}$$

where ***I***_*p*_ is a *p* × *p* identity matrix, and the symmetric matrix $({\Vert {{\boldsymbol{s}}}_{r}\Vert }_{2}^{2}{{\boldsymbol{I}}}_{p}+{\lambda }_{L}{\boldsymbol{L}})$ (*r* = 1 in this case) is an invertible matrix (see Supplementary materials for detailed proof). Subsequently, the gene score vector and sample indicator vector in the first component can be iteratively estimated through alternating the two update rules (5) and (7) until convergence^36, 37, 40^.

**Algorithm 1.** The iterative estimation procedure of sample indicator vector and gene score vector in mCGfinder.

**Algorithm 1** mCGfinder: iterative estimation procedure
**Input:** mutation matrix ${{\boldsymbol{X}}}_{n\times p}$; graph Laplacian matrix ***L***;
**Output:** sample indicator vector **s**_1_; gene score vector ***g***_1_.
1: set *λ*_*L*_ ← 0.1
2: $${{\boldsymbol{s}}}_{1}^{\mathrm{(0)}}\leftarrow {{\bf{1}}}_{n}$$ and $${{\boldsymbol{g}}}_{1}^{\mathrm{(0)}}\leftarrow {(n{{\boldsymbol{I}}}_{p}+{\lambda }_{L}{\boldsymbol{L}})}^{-1}({{\boldsymbol{X}}}^{{\rm{T}}}{{\bf{1}}}_{n})$$
3: **repeat**
4: $${{\boldsymbol{g}}}_{1}^{(k+\mathrm{1)}}\leftarrow {({\Vert {{\boldsymbol{s}}}_{1}^{(k)}\Vert }_{2}^{2}{{\boldsymbol{I}}}_{p}+{\lambda }_{L}{\boldsymbol{L}})}^{-1}({{\boldsymbol{X}}}^{{\rm{T}}}{{\boldsymbol{s}}}_{1}^{(k)})$$
6: $${{\boldsymbol{s}}}_{1}^{(k+\mathrm{1)}}\leftarrow {{\boldsymbol{I}}}_{[\mathrm{0,}\infty )}(2{\boldsymbol{X}}{{\boldsymbol{g}}}_{1}^{(k+\mathrm{1)}}-{\Vert {{\boldsymbol{g}}}_{1}^{(k+\mathrm{1)}}\Vert }_{2}^{2})$$
6: *k* ← *k* + 1
7: **until** Convergence
8: **return** $${{\boldsymbol{s}}}_{1}\leftarrow {{\boldsymbol{s}}}_{1}^{(\infty )}$$ and $${{\boldsymbol{g}}}_{1}\leftarrow {{\boldsymbol{g}}}_{1}^{(\infty )}$$
**Note:** **1**_*n*_ is an *n* × 1 vector with all coefficients being 1; Indicator function ***I***_***A***_(***x***) returns a logical vector if *x*_*i*_ ∈ ***A***.

The algorithm of the estimation of the two vectors in the first component are summarized in Algorithm 1.

After convergence, the first component is obtained by matrix multiplication ${{\boldsymbol{s}}}_{1}{{\boldsymbol{g}}}_{1}^{{\rm{T}}}$. To obtain the next component (***s***_2_ and ***g***_2_), we repeat the procedures in Algorithm 1 on the remaining samples^36, 37, 40^. Subsequently, we can estimate the *r*-th component (***s***_*r*_ and ***g***_*r*_) (*r* = 2, …, *R*) by decomposing the data matrix through the component-by-component strategy until all samples are assigned (details in Supplementary Fig. S7), and the number *R* is obtained by counting the components decomposed by mCGfinder.

### Significance test

To assess which of these mutated genes are statistically significant in a subset of samples, we implement significance test on the coefficients of the gene score vectors ***g***_*r*_ (*r* = 1, …, *R*) in every components decomposed by mCGfinder. In brief, we define ${\boldsymbol{X}}{({\Vert {{\boldsymbol{s}}}_{r}\Vert }_{2}^{2}{{\boldsymbol{I}}}_{p}+{\lambda }_{L}{\boldsymbol{L}})}^{-1}$ in (7) as the network influenced matrix. The coefficients of gene score vector ***g***_*r*_ can be calculated by the summation of the entries of a subset of rows of the network influenced matrix ***X***_net_, where the rows are indicated by the sample indicator vector ***s***_*r*_ of the investigated component. We follow the procedure in previous studies^40, 57^ and identify the genes of which the scores can disprove the null hypothesis that their values of the gene score vector coefficients are only contributed by background mutations alone. Since the random background mutations could occur anywhere in the genome, the null distribution is modeled by recalculating the gene score vectors across all combinations of permutations of the network influenced matrix within samples. Detailed procedure for the significance test is provided in Supplementary materials. Since large numbers of permutations is usually time consuming, we instead use a semi-exact estimation approach proposed in previous approaches^40, 57^ to estimate the distribution of scores and the corresponding p-values. To control the false discovery rates of the investigated genes, we apply the Benjamini-Hochberg FDR procedure^58^ on the p-values obtained from the significance test, and calculate the q-values of the investigated genes for each component. For a specific gene, we choose the most significant (smallest) q-values of the investigated gene among all components as the significance score of the gene.

## Electronic supplementary material

Supplementary Materials
