# Supplementary material for: A novel network regularized matrix decomposition method to detect mutated cancer genes in tumour samples with inter-patient heterogeneity
Source: Sci Rep. 2017 Jun 6;7:2855. doi: 10.1038/s41598-017-03141-w (PMC5460199; doi:10.1038/s41598-017-03141-w)
Supplement: Supplementary file 1 — Supplementary Materials [file 41598_2017_3141_MOESM1_ESM.pdf]

Supplementary Materials:

A novel network regularized matrix decomposition method to detect mutated cancer genes in tumour samples with inter-patient heterogeneity

Jianing Xi [1,+], Ao Li [1,2,+,*] and Minghui Wang [1,2]

[1]School of Information Science and Technology, University of Science and Technology of China, Hefei AH230027, China and
[2]Centers for Biomedical Engineering, University of Science and Technology of China, Hefei AH230027, China.
[*]E-mail: aoli@ustc.edu.cn
[+]These authors contributed equally to this work

Contents

1 Supplementary Material **2**
- 1.1 Configuration settings of mCGfinder, HotNet2 and ReMIC . . . 2
- 1.2 Ranking of the genes detected by mCGfinder, HotNet2 and ReMIC 2
- 1.3 Normalized Adjacency and Laplacian matrix normalization . . . 2
- 1.4 Proof: $\left(\|\boldsymbol{s}_r\|_2^2\,\mathbf{I}_p + \lambda_L \mathbf{L}\right)$ is an invertible matrix 3
- 1.5 Significance test through a semiexact estimation 3
- 1.6 Input data: TCGA somatic mutation data 4

2 Supplementary Figures and Captions **6**

3 Supplementary Tables and Captions **13**

1 Supplementary Material

1.1 Configuration settings of mCGfinder, HotNet2 and ReMIC

In comparison analysis, the detection results of mCGfinder are selected by default threshold 0.05. The tuning parameter used to balance the fitness of the model and the smoothness of the scores of connected genes is set to 0.1.

In HotNet2, the parameter of insulated heat-diffusion β is set to 0.45 for the iRefIndex network [1] as suggested [2]. The permuted networks of the gene interaction network are generated by HotNet2. The scores of the genes are set to their mutation frequencies, and the numbers of delta permutations and significance permutations are set to 100 as default settings [2].

In ReMIC, the number of iterations to generate the bag of random mutation score is set to 20000, which satisfy the condition that it is larger than number of genes (totally 12129 genes in iRefIndex network). The number of permutations is set to 10000. The pseudocount is set to true. The diffusion strength β is set to 0.01, 0.02 and 0.03 respectively as suggested in ReMIC [3] (scale parameter of 0 is excluded since its result is equivalent to gene mutation frequencies). Since the performance of ReMIC with $\beta = 0.03$ is relative better than the performance with $\beta = 0.01$ or 0.02, we use the results of ReMIC with $\beta = 0.03$ in the comparison study.

1.2 Ranking of the genes detected by mCGfinder, HotNet2 and ReMIC

The genes detected by mCGfinder are ranked by negative logarithm of q-values. The q-values of genes are calculated by the significance test and false discovery rate control [4] in mCGfinder.

The genes detected by HotNet2 are ranked by the values of the minimum edge weight parameter δ, which have been used to calculate the true positive rates and false positive rates in previous study [2].

The genes detected by ReMIC are sorted by the negative logarithm of p-values, which are calculated by permutation test in ReMIC [3].

1.3 Normalized Adjacency and Laplacian matrix normalization

The gene interaction network in iRefindex [1] is an undirected, unweighted graph $G = (\boldsymbol{V}, \boldsymbol{E})$ without graph loops (i, i) or multiple edges from one node to another, where $\boldsymbol{V}$ is the vertex set, $p = |\boldsymbol{V}|$, and $\boldsymbol{E}$ is the edge set. Then the symmetric normalized adjacency matrixof the graph G is an $p \times p$ symmetric matrix with one row and column for each node defined by [5]:

$$\boldsymbol{A} = \boldsymbol{D}^{-1/2} \boldsymbol{A}^{(adj)} \boldsymbol{D}^{-1/2},$$

where $\boldsymbol{D} = \mathrm{Diag}(d_1, \ldots, d_i, \ldots, d_p)$ for d_i the degree of node i in the graph G and $\boldsymbol{A}^{(adj)}$ is the original adjacency matrix of graph G. Therefore, the diagonal

elements A_{ij} of $\boldsymbol{A}$ are equal the elements $A_{ij}^{(adj)}$ of $\boldsymbol{A}^{adj}$ divided by the square root of the product of d_i and d_j, i.e.

$$A_{ij} = A_{ij}^{adj} / \sqrt{d_i d_j}. \tag{1}$$

The normalized Laplacian Matrix is

$$\boldsymbol{L} = \boldsymbol{I} - \boldsymbol{A} = \boldsymbol{D}^{-1/2}(\boldsymbol{D} - \boldsymbol{A}_{adj})\boldsymbol{D}^{-1/2} = \boldsymbol{D}^{-1/2}\boldsymbol{L}_G\boldsymbol{D}^{-1/2},$$

for $\boldsymbol{L}_G$ the un-normalized Laplacian. Therefore, the diagonal elements L_{ij} of $\boldsymbol{L}$ are equal the degree of vertex i and off-diagonal elements L_{ij} are -1 if vertex i is adjacent to j and 0 otherwise [5], i.e.

$$L_{ij} = \begin{cases} 1 & \text{if } i = j \text{ and } d_j \neq 0, \\ -\frac{1}{\sqrt{d_i d_j}} & \text{if } i \text{ and } j \text{ are adjacent}, \\ 0 & \text{otherwise.} \end{cases} \tag{2}$$

1.4 Proof: $\left(\|\boldsymbol{s}_r\|_2^2 \mathbf{I}_p + \lambda_L \mathbf{L}\right)$ is an invertible matrix

Proof. Note that graph Laplacian matrix $\mathbf{L}$ ($p \times p$) is positive semidefinite. Thus, through eigendecomposition, it can be factorized as $\mathbf{L} = \mathbf{P}^{\mathrm{T}}\boldsymbol{\Lambda}\mathbf{P}$, where $\mathbf{P}$ is an orthogonal matrix and $\boldsymbol{\Lambda}$ is a diagonal matrix whose diagonal entries are the eigenvalues of $\mathbf{L}$. Due to positive semidefinite, all diagonal entries of diagonal matrix $\boldsymbol{\Lambda}$ are nonnegative.

Because of the matrix orthogonality $\mathbf{P}^{\mathrm{T}}\mathbf{P} = \mathbf{I}_p$, the matrix $\left(\|\boldsymbol{s}_r\|_2^2 \mathbf{I}_p + \lambda_L \mathbf{L}\right)$ can be factorized as

$$\left(\|\boldsymbol{s}_r\|_2^2 \mathbf{I}_p + \lambda_L \mathbf{L}\right) = \mathbf{P}^{\mathrm{T}} \left(\|\boldsymbol{s}_r\|_2^2 \mathbf{I}_p + \lambda_L \boldsymbol{\Lambda}\right) \mathbf{P},$$

where $\left(\|\boldsymbol{s}_r\|_2^2 \mathbf{I}_p + \lambda_L \boldsymbol{\Lambda}\right)$ is also a diagonal matrix. Note that $\|\boldsymbol{s}_r\|_2^2$ is always positive, λ_L is an nonnegative tuning parameter and all diagonal entries of matrix $\boldsymbol{\Lambda}$ are nonnegative. Consequently, all diagonal entries of $\left(\|\boldsymbol{s}_r\|_2^2 \mathbf{I}_p + \lambda_L \boldsymbol{\Lambda}\right)$ are positive, suggesting that it is a positive definite matrix. Therefore, the investigated matrix $\left(\|\boldsymbol{s}_r\|_2^2 \mathbf{I}_p + \lambda_L \mathbf{L}\right)$ is invertible.

1.5 Significance test through a semiexact estimation

In brief, we define $\mathrm{X}_{\mathrm{net}} := \left[\left(\|\boldsymbol{s}_r\|_2^2 \mathrm{I}_p + \lambda_L \mathrm{L}\right)^{-1} \mathrm{X}^{\mathrm{T}}\right]^{\mathrm{T}} = \mathrm{X}\left(\|\boldsymbol{s}_r\|_2^2 \mathrm{I}_p + \lambda_L \mathrm{L}\right)^{-1}$ as the network influenced matrix. For the r-th component, the coefficients of gene score vector $\boldsymbol{g}_r$ can be calculated by the summation of the entries of a subset of rows of the network influenced matrix $\mathrm{X}_{\mathrm{net}}$, where the rows are indicated by the sample indicator vector $\boldsymbol{s}_r$ of the investigated component. To assess which of these mutated genes are statistically significant in a subset of samples, we follow the procedure of previous studies [6, 7] and identify the genes of which the scores can disprove the null hypothesis that their values of the gene score vector coefficients are only contributed by background mutations alone. Since the random background mutations could occur anywhere in the genome,

we model the null distribution by recalculating the gene score vectors across all combinations of permutations of the network influenced matrix X_{net} within rows (samples) indicated by $\boldsymbol{s}_r$ of the r-th component [6, 7]. Under the null hypothesis, the arrangement of entries in X_{net} is independent between the indicated samples (rows). Accordingly, by permuting the entries in the rows indicated by $\boldsymbol{s}_r$ of the matrix X_{net}, we can generate a conservative, high estimate of the null distribution which contains information from both the somatic mutations and the network context.

Since large numbers of permutations is usually time consuming, we follow the procedure proposed in previous approaches [6, 7] by using a semi-exact estimate of this null distribution instead of simulating the null distribution by performing each of these permutations in turn. The distribution of the sum of across the indicated rows equals the convolution of the distributions of entries in all the indicated rows. For the investigated component, we approximate these distributions by generating histograms $\boldsymbol{h}_i^r(x_{\text{net}})$ for the i-th indicated row of the network influenced matrix X_{net}, where the number of bins is set to 10^5. The final distribution for coefficient values of the gene score vector is calculated by $\boldsymbol{H}^r = \boldsymbol{h}_1^r \otimes \boldsymbol{h}_2^r \ldots \otimes \boldsymbol{h}_{l_r}^r$, where $1, 2, \ldots, l_r$ is the indices of rows indicated by the r-th component (totally l_r samples included in the investigated component). By comparing the coefficients of the estimated vector $\boldsymbol{g}_r$ to the distribution above, we can assign the p-value for each investigated gene by the sum of the tail of the null distribution estimated above.

1.6 Input data: TCGA somatic mutation data

In this study, we use TCGA somatic mutation data to evaluate the performances of mCGfinder. To prevent mutagens or carcinogens involved in cancer treatment which could cloud the origin of the cancer, TCGA have strict sample criteria in acquiring tissue samples, such as "sample from primary tumor was necessary" and "neoadjuvant treatment was not allowable":
https://cancergenome.nih.gov/cancersselected/biospeccriteria
Therefore, to the best of our knowledge, the underlying genomics of primary, untreated tumor samples in TCGA is not affected by chemotherapy.

In consistent with HotNet2 and ReMIC, somatic mutation data are required as the input of mCGfinder. Therefore, somatic mutations from raw data files should be filtered to remove polymorphisms as described in previous study [8]. More detailed information of the datasets of the investigated cancers are provided in Supplementary Table S5.

References

[1] Sabry Razick, George Magklaras, and Ian M Donaldson. irefindex: a consolidated protein interaction database with provenance. *BMC bioinformatics*, 9(1):1, 2008.

[2] Mark D Leiserson, Fabio Vandin, Hsin-Ta Wu, Jason R Dobson, and Benjamin R Raphael. Pan-cancer identification of mutated pathways and protein complexes. *Cancer Research*, 74(19 Supplement):5324–5324, 2014.

[3] Sepideh Babaei, Marc Hulsman, Marcel Reinders, and Jeroen de Ridder. Detecting recurrent gene mutation in interaction network context using multi-scale graph diffusion. *BMC bioinformatics*, 14(1):1, 2013.

[4] Yoav Benjamini and Yosef Hochberg. Controlling the false discovery rate: a practical and powerful approach to multiple testing. *Journal of the royal statistical society. Series B (Methodological)*, pages 289–300, 1995.

[5] Fan RK Chung. *Spectral graph theory*, volume 92. American Mathematical Soc., 1997.

[6] Jianing Xi and Ao Li. Discovering recurrent copy number aberrations in complex patterns via non-negative sparse singular value decomposition. *IEEE/ACM Transactions on Computational Biology and Bioinformatics*, 13(4):656–668, 2016.

[7] Rameen Beroukhim, Gad Getz, Leia Nghiemphu, Jordi Barretina, Teli Hsueh, David Linhart, Igor Vivanco, Jeffrey C Lee, Julie H Huang, Sethu Alexander, et al. Assessing the significance of chromosomal aberrations in cancer: methodology and application to glioma. *Proceedings of the National Academy of Sciences*, 104(50):20007–20012, 2007.

[8] Cancer Genome Atlas Network et al. Comprehensive molecular portraits of human breast tumours. *Nature*, 490(7418):61–70, 2012.

[9] P Andrew Futreal, Lachlan Coin, Mhairi Marshall, Thomas Down, Timothy Hubbard, Richard Wooster, Nazneen Rahman, and Michael R Stratton. A census of human cancer genes. *Nature Reviews Cancer*, 4(3):177–183, 2004.

[10] Abel Gonzalez-Perez, Christian Perez-Llamas, Jordi Deu-Pons, David Tamborero, Michael P Schroeder, Alba Jene-Sanz, Alberto Santos, and Nuria Lopez-Bigas. Intogen-mutations identifies cancer drivers across tumor types. *Nature methods*, 10(11):1081–1082, 2013.

[11] Jingchun Zhu, J Zachary Sanborn, Stephen Benz, Christopher Szeto, Fan Hsu, Robert M Kuhn, Donna Karolchik, John Archie, Marc E Lenburg, Laura J Esserman, et al. The ucsc cancer genomics browser. *Nature methods*, 6(4):239–240, 2009.

2 Supplementary Figures and Captions

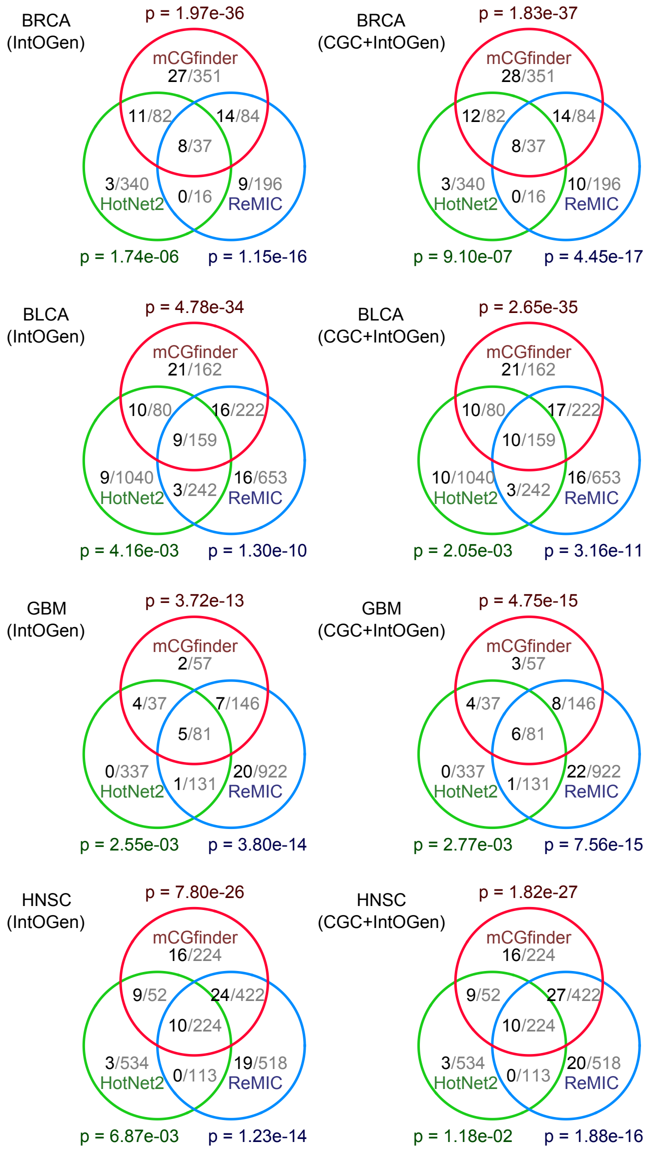

Supplementary Figure S1. Venn diagrams of intersections between the genes detected by mCGfinder (red circle), HotNet2 (green circle) and ReMIC (blue circle) on TCGA somatic mutation datasets of BRCA (first row), BLCA (second row), GBM (third row) and HNSC (fourth row). In each region, The gray and black numbers in each region of the Venn diagrams indicate the number of detected genes and the number of genes also reported in IntOGen gene lists (first column) and the combined lists of the two databases (second column) respectively. The p-values next to the circles of methods are the enrichment significance of the detection results for the validation known cancer gene lists.

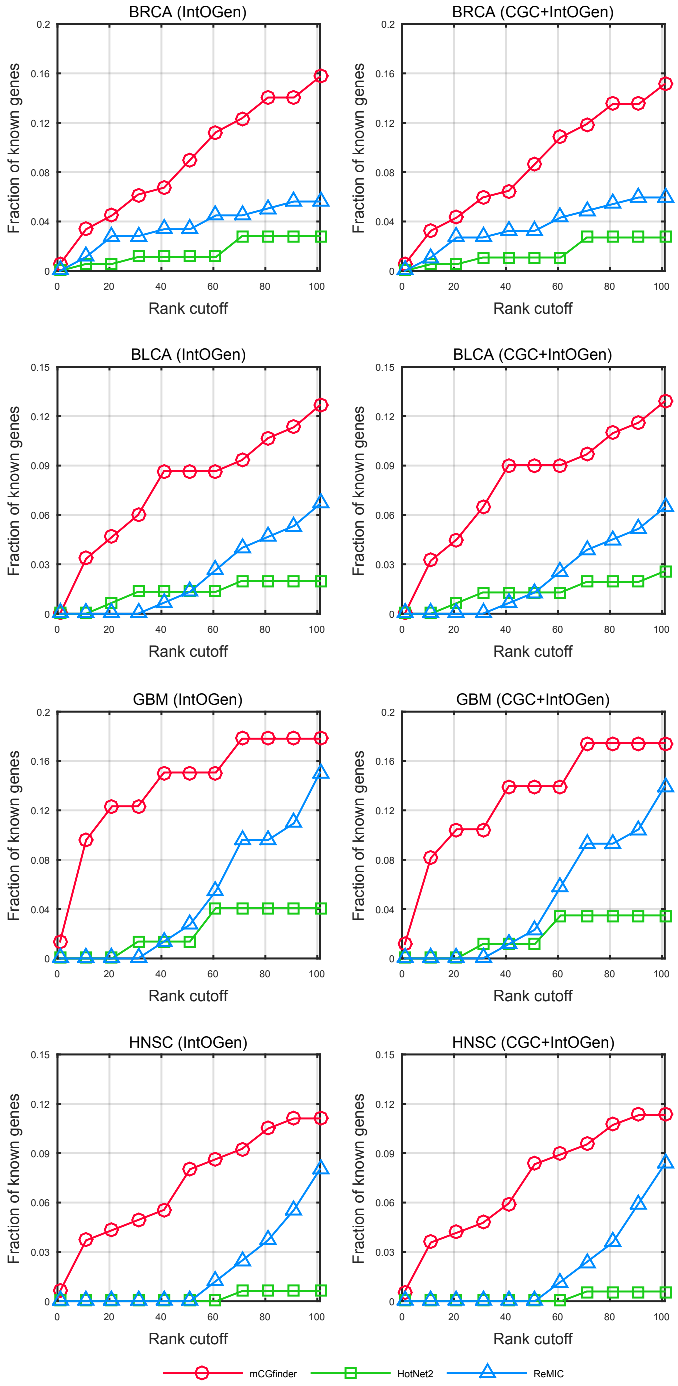

Supplementary Figure S2. Rank cutoff curves of top 100 candidates in mCGfinder (red line with circle markers), HotNet2 (green line with square markers) and ReMIC (blue line with triangle markers) results, describing the relation between various cutoffs and the fraction of known IntOGen cancer genes (first column) and the combined genes from the two databases (second column) that are ranked above this cutoff in BRCA (first row), BLCA (second row), GBM (third row) and HNSC (fourth row).

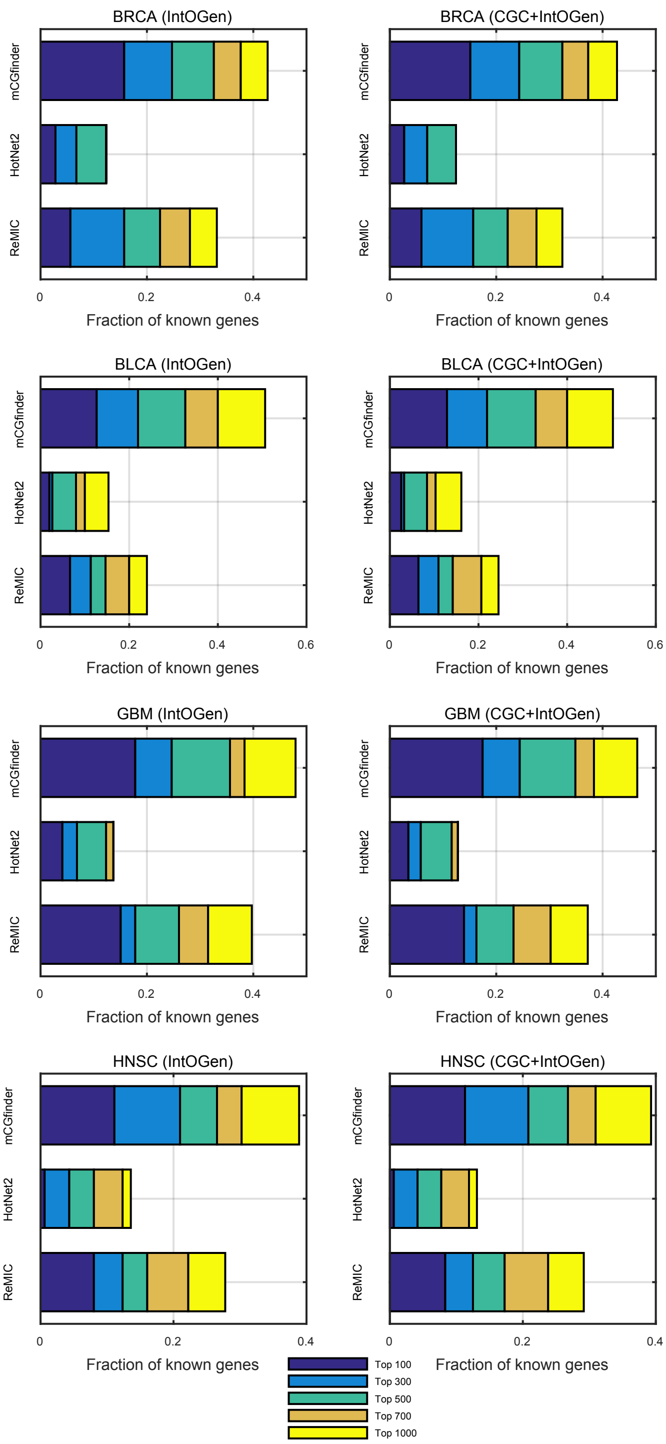

Supplementary Figure S3. Cumulative fractions of known cancer genes reported in IntOGen (first column) and the combined genes lists from the two databases (second column) within the top 100, 300, 500, 700 and 1000 genes on BRCA (first row), BLCA (second row), GBM (third row) and HNSC (fourth row).

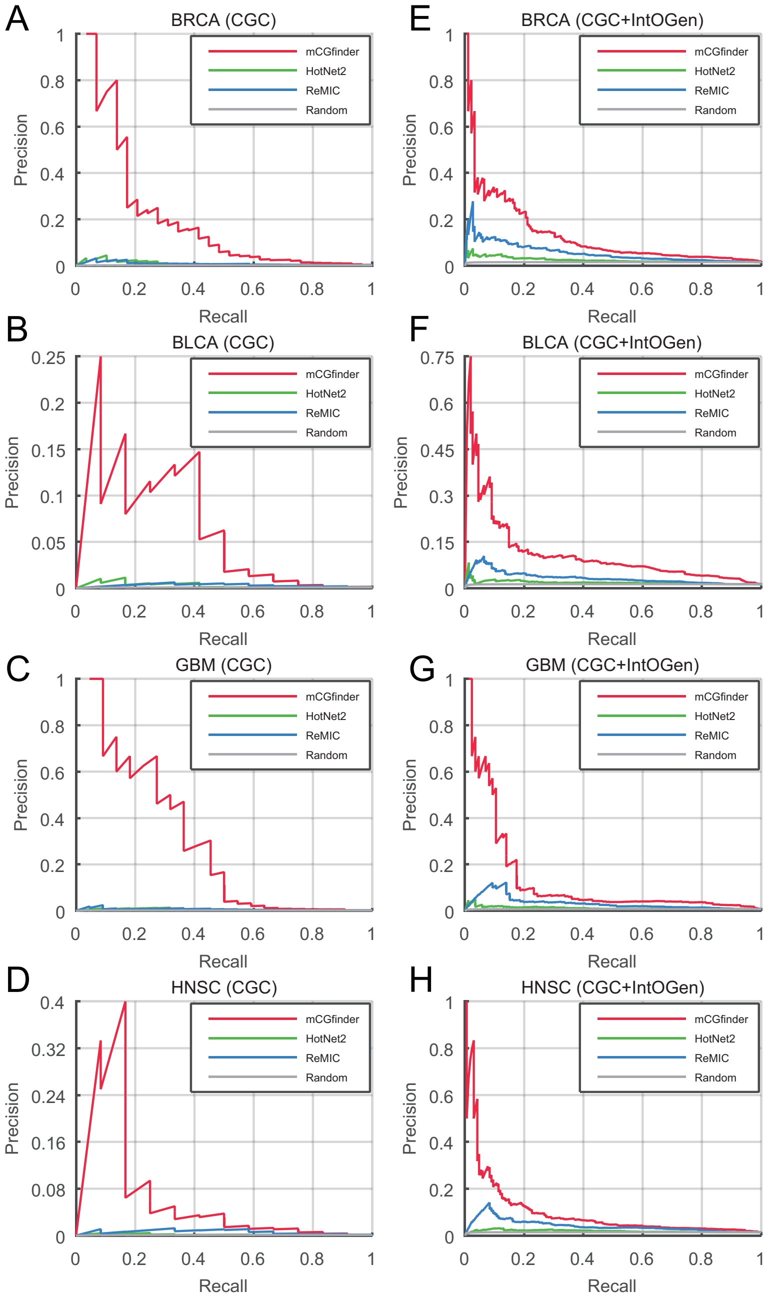

Supplementary Figure S4. Precision-recall curves for the three methods on BRCA (A and E), BLCA (B and F), GBM (C and G) and HNSC (D and H) data, validated by CGC (A-D) and the combined genes lists from both CGC and IntOGen (E-H) respectively. where each point indicates the precision and recall at a different rank in the prediction. Red, green and blue lines represent the curves of mCGfinder, HotNet2 and ReMIC results.

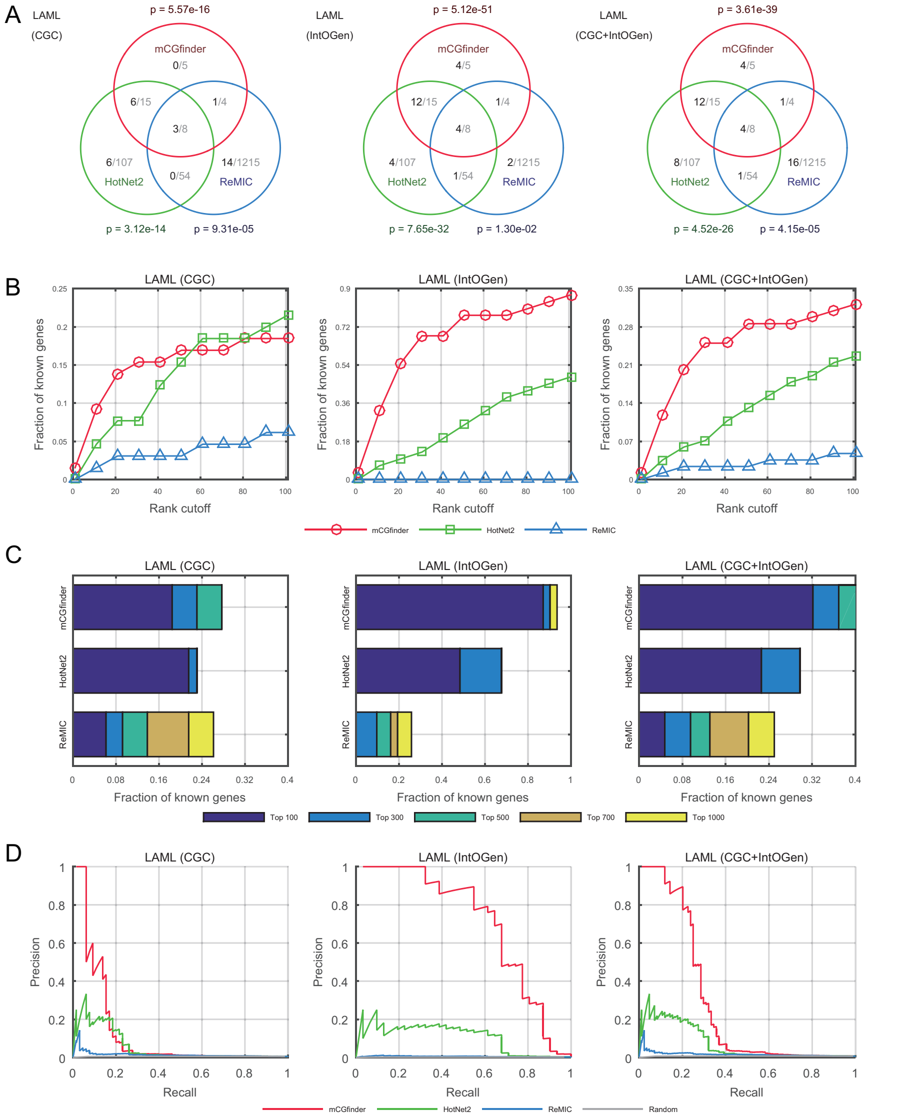

Supplementary Figure S5. The detection results on TCGA LAML data given by mCGfinder (red), HotNet2 (green) and ReMIC (blue), validated by CGC (left column), IntOGen (middle column) and the combined genes lists from the two databases (right column). (A) Venn diagrams of intersections between the genes detected by the three methods. (B) Rank cutoff curves of top 100 candidates detected by the three methods. (C) Cumulative fractions of known cancer genes within the top 100, 300, 500, 700 and 1000 genes. (D) Precision-recall curves for the three methods.

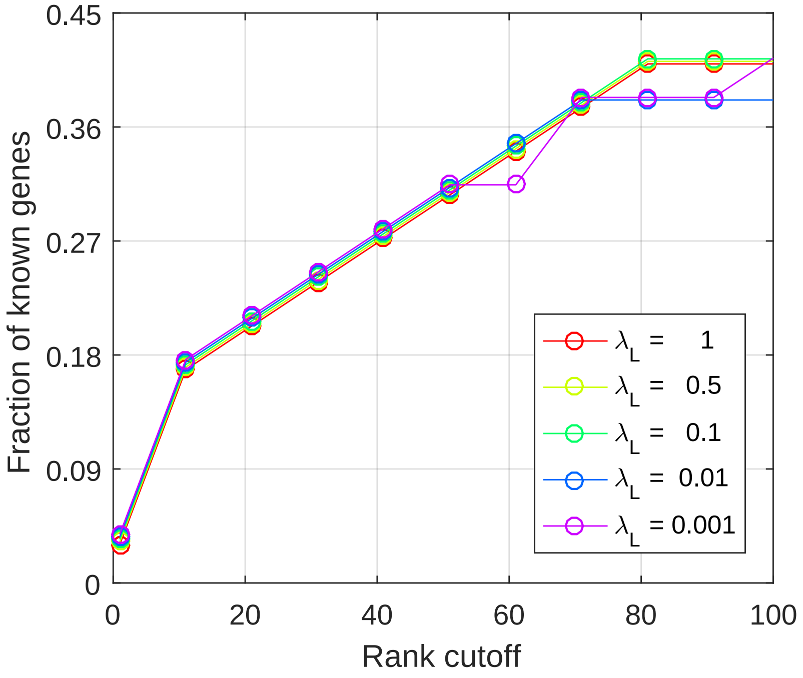

Supplementary Figure S6. The detection results of mCGfinder with different tuning parameter values on BRCA data. The results show that the performance of mCGfinder is not sensitive to the selection of the tuning parameter when the value varies from 1 to 0.001.

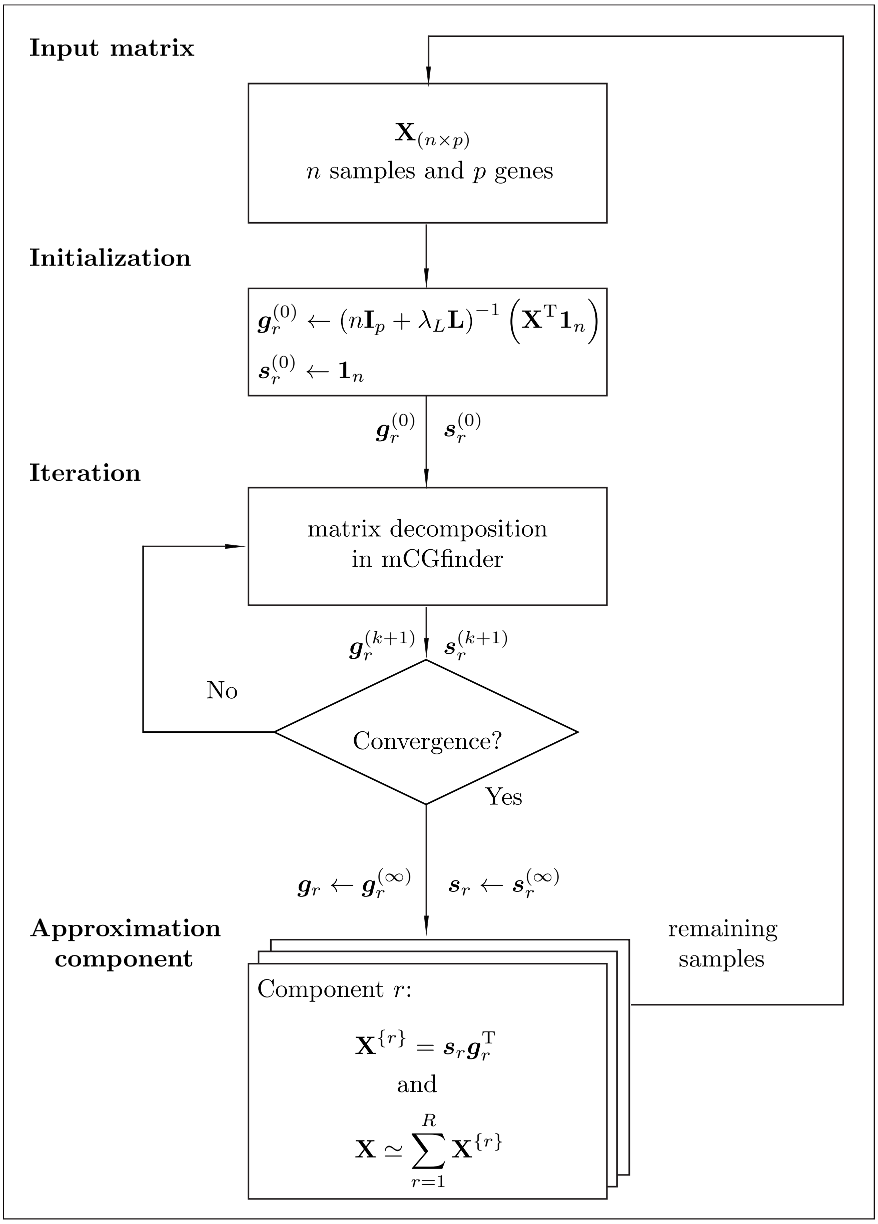

Supplementary Figure S7. Flowchart of the component-by-component decomposition strategy in mCGfinder model. After initialization, mCGfinder estimates the sample indicator vector $\boldsymbol{s}$ and the gene score vector $\boldsymbol{g}$ through the iterative estimation procedure until convergence. Then the component can be obtained through the outer product of the two vectors $\boldsymbol{s}\boldsymbol{g}^{\mathrm{T}}$. We repeat this procedures aforementioned on the remaining samples, until all samples are assigned.

3 Supplementary Tables and Captions

Supplementary Table S1. The full lists of Cancer Gene Census (CGC) annotated cancer genes [9] detected by mCGfinder on BRCA (A), BLCA (B), GBM (C), HNSC (D) and LAML (E) data, sorted by their rank in mCGfinder result.

(A) CGC genes in BRCA results

Gene Symbol	Rank	q-value	also detected by
PIK3CA	1	$\leq$1.00e-159	HotNet2/ReMIC
TP53	2	$\leq$1.00e-159	HotNet2/ReMIC
GATA3	4	1.96e-99	HotNet2
MAP3K1	5	2.19e-44	
CDH1	9	1.18e-28	HotNet2
NCOR1	21	1.03e-11	
MAP2K4	29	2.87e-10	HotNet2
CTCF	32	2.04e-08	
AKT1	45	8.21e-07	
RB1	53	1.64e-06	ReMIC
ARID1A	69	1.44e-05	HotNet2
FOXA1	73	4.48e-05	HotNet2
TBX3	104	2.57e-04	
ARID1B	154	2.67e-03	HotNet2
BRCA2	240	1.10e-02	
ERBB2	340	1.82e-02	ReMIC
CASP8	392	4.03e-02	
MAP3K13	456	4.03e-02	

(B) CGC genes in BLCA results

Gene Symbol	Rank	q-value	also detected by
KDM6A	4	6.80e-41	HotNet2
STAG2	12	3.94e-17	HotNet2/ReMIC
LRP1B	26	1.76e-11	ReMIC
FGFR3	30	2.01e-10	HotNet2
ERBB3	34	2.07e-09	ReMIC
TSC1	96	9.24e-06	mCGfinder
NOTCH2	340	6.68e-03	HotNet2/ReMIC
NOTCH1	537	2.40e-02	ReMIC

(C) CGC genes in GBM results

Gene Symbol	Rank	q-value	also detected by
PTEN	1	1.99e-159	HotNet2
TP53	2	7.24e-141	HotNet2
EGFR	4	1.42e-109	HotNet2/ReMIC
PIK3CA	6	3.70e-38	HotNet2/ReMIC

continued on next page

continued from previous page

PIK3R1	8	4.14e-33	HotNet2/ReMIC
NF1	9	1.80e-31	ReMIC
ATRX	14	2.12e-17	ReMIC
IDH1	17	3.59e-13	HotNet2
PDGFRA	32	4.51e-08	ReMIC
STAG2	33	4.51e-08	ReMIC
LZTR1	66	7.43e-05	
ROS1	283	2.36e-02	HotNet2/ReMIC

(D) CGC genes in HNSC results

Gene Symbol	Rank	q-value	also detected by
FAT1	3	6.39e-114	ReMIC
NOTCH1	5	1.54e-78	HotNet2/ReMIC
FAT4	32	3.46e-16	ReMIC
NFE2L2	80	2.45e-09	HotNet2/ReMIC
TGFBR2	144	8.53e-07	
CTCF	160	4.80e-06	HotNet2
ERBB3	421	2.33e-03	ReMIC
BCORL1	615	1.07e-02	ReMIC
MTOR	753	2.95e-02	ReMIC

(E) CGC genes in LAML results

GeneSymbol	Rank	q-value	also detected by
NPM1	1	5.18e-165	mCGfinder/HotNet2
DNMT3A	2	1.03e-93	mCGfinder/HotNet2/ReMIC
FLT3	3	4.11e-87	mCGfinder/ReMIC
RUNX1	4	2.22e-45	mCGfinder/HotNet2
PTPN11	9	5.07e-10	mCGfinder/HotNet2/ReMIC
NRAS	10	1.13e-09	mCGfinder/HotNet2
CEBPA	15	3.79e-06	mCGfinder/HotNet2
KIT	16	4.17e-06	mCGfinder/HotNet2
RAD21	17	7.77e-05	mCGfinder/HotNet2
KRAS	23	1.45e-03	mCGfinder/HotNet2/ReMIC

Supplementary Table S2. The full lists of Integrative Onco Genomics (IntOGen) annotated cancer genes [10] detected by mCGfinder on BRCA (A), BLCA (B), GBM (C), HNSC (D) and LAML (E) data, sorted by their rank in mCGfinder result.

(A) IntOGen genes in BRCA results

Gene Symbol	Rank	q-value	also detected by
PIK3CA	1	leq1.00e-159	HotNet2/ReMIC
TP53	2	leq1.00e-159	HotNet2/ReMIC
GATA3	4	1.96e-99	HotNet2
MAP3K1	5	2.19e-44	
MLL3	8	2.45e-34	HotNet2/ReMIC
CDH1	9	1.18e-28	HotNet2
MACF1	20	1.03e-11	HotNet2/ReMIC
NCOR1	21	1.03e-11	
PTEN	27	1.34e-10	HotNet2/ReMIC
CBFB	28	2.87e-10	HotNet2
MAP2K4	29	2.87e-10	HotNet2
CTCF	32	2.04e-08	
RUNX1	44	2.82e-07	
AKT1	45	8.21e-07	
ASPM	46	1.64e-06	HotNet2
NF1	51	1.64e-06	ReMIC
PIK3R1	52	1.64e-06	ReMIC
RB1	53	1.64e-06	ReMIC
ATM	58	4.76e-06	
AKAP9	60	1.37e-05	
ARID1A	69	1.44e-05	HotNet2
TBL1XR1	71	2.58e-05	
FOXA1	73	4.48e-05	HotNet2
ANK3	76	8.87e-05	HotNet2
ASH1L	77	8.87e-05	
MYH14	92	8.87e-05	HotNet2
SETDB1	95	8.87e-05	
SVEP1	96	8.87e-05	ReMIC
SF3B1	102	1.07e-04	
TBX3	104	2.57e-04	
CCAR1	113	5.16e-04	HotNet2
MLL2	128	5.16e-04	HotNet2/ReMIC
RBM5	137	5.16e-04	
RPGR	139	5.16e-04	HotNet2
BRCA1	157	2.67e-03	ReMIC
CAD	159	2.67e-03	
CHD4	162	2.67e-03	
MGA	186	2.67e-03	
AHNAK	224	1.10e-02	ReMIC
BRCA2	240	1.10e-02	
EGFR	262	1.10e-02	ReMIC
MTOR	285	1.10e-02	

continued on next page

continued from previous page

MYH11	288	1.10e-02	ReMIC
NOTCH2	298	1.10e-02	ReMIC
SETD2	318	1.10e-02	HotNet2/ReMIC
STAG2	320	1.10e-02	HotNet2/ReMIC
TAF1	322	1.10e-02	
HCFC1	337	1.16e-02	
ERBB2	340	1.82e-02	ReMIC
PIK3CB	346	2.45e-02	
KRAS	354	3.19e-02	
MYB	355	3.19e-02	
ZFP36L1	370	3.63e-02	
ATR	384	4.03e-02	ReMIC
CASP8	392	4.03e-02	
MLL	462	4.03e-02	
MLLT4	464	4.03e-02	ReMIC
MYH9	466	4.03e-02	
SMARCA4	513	4.03e-02	ReMIC
FN1	553	4.83e-02	ReMIC

(B) IntOGen genes in BLCA results

Gene Symbol	Rank	q-value	also detected by
TP53	2	5.81e-116	
ARID1A	3	7.86e-44	HotNet2/ReMIC
KDM6A	4	6.80e-41	HotNet2
RB1	7	3.34e-26	
ELF3	11	3.94e-17	HotNet2
STAG2	12	3.94e-17	HotNet2/ReMIC
EP300	15	5.55e-16	HotNet2
CDKN1A	29	2.01e-10	
FGFR3	30	2.01e-10	HotNet2
ERBB3	34	2.07e-09	ReMIC
ERCC2	35	2.07e-09	
FAT1	36	2.07e-09	ReMIC
AHNAK	41	2.10e-08	ReMIC
NCOR2	64	1.41e-06	ReMIC
ARHGAP35	73	9.24e-06	HotNet2/ReMIC
FBXW7	78	9.24e-06	HotNet2/ReMIC
HSP90AA1	83	9.24e-06	
TRIO	94	9.24e-06	ReMIC
TSC1	96	9.24e-06	
ANK3	102	5.70e-05	HotNet2/ReMIC
CHEK2	105	5.70e-05	
NFE2L2	115	5.70e-05	HotNet2/ReMIC
TP53BP1	176	3.13e-04	ReMIC
TXNIP	178	3.13e-04	HotNet2
APC	184	5.49e-04	ReMIC
ATR	186	5.49e-04	ReMIC

continued on next page

continued from previous page

CDKN2A	204	1.47e-03	ReMIC
MGA	236	1.47e-03	ReMIC
MYH10	242	1.47e-03	
RHOA	257	1.47e-03	ReMIC
SETD2	261	1.47e-03	HotNet2/ReMIC
SF3B1	262	1.47e-03	
CAD	300	6.68e-03	ReMIC
CNOT1	304	6.68e-03	HotNet2
HLA-A	326	6.68e-03	HotNet2
NUP98	341	6.68e-03	
SMC1A	357	6.68e-03	
CLTC	384	1.64e-02	
FN1	391	1.64e-02	ReMIC
CLSPN	423	1.77e-02	
HSP90AB1	429	1.87e-02	
ARID1B	430	1.91e-02	HotNet2/ReMIC
ACTB	438	2.40e-02	
AFF4	441	2.40e-02	HotNet2
CDK12	461	2.40e-02	HotNet2/ReMIC
CHD3	464	2.40e-02	
CHD9	465	2.40e-02	ReMIC
CLASP2	466	2.40e-02	HotNet2
EIF2AK3	481	2.40e-02	HotNet2
MAP3K1	520	2.40e-02	
MAP3K4	521	2.40e-02	ReMIC
MECOM	524	2.40e-02	
MLH1	527	2.40e-02	
NAP1L1	532	2.40e-02	
NOTCH1	537	2.40e-02	ReMIC
PTEN	556	2.40e-02	

(C) IntOGen genes in GBM results

Gene Symbol	Rank	q-value	also detected by
PTEN	1	1.99e-159	HotNet2
TP53	2	7.24e-141	HotNet2
EGFR	4	1.42e-109	HotNet2/ReMIC
PIK3CA	6	3.70e-38	HotNet2/ReMIC
PIK3R1	8	4.14e-33	HotNet2/ReMIC
NF1	9	1.80e-31	ReMIC
RB1	11	2.84e-28	
ATRX	14	2.12e-17	ReMIC
IDH1	17	3.59e-13	HotNet2
STAG2	33	4.51e-08	ReMIC
CHD8	36	5.77e-07	HotNet2/ReMIC
KDR	64	7.43e-05	HotNet2/ReMIC
RPL5	68	7.43e-05	
PTPN11	158	4.59e-03	ReMIC

continued on next page

continued from previous page

AKAP9	184	2.36e-02	ReMIC
BRAF	194	2.36e-02	ReMIC
BRCA1	195	2.36e-02	ReMIC
CLOCK	202	2.36e-02	HotNet2

(D) IntOGen genes in HNSC results

Gene Symbol	Rank	q-value	also detected by
TP53	1	¡=1.00e-159	HotNet2
FAT1	3	6.39e-114	ReMIC
CDKN2A	4	9.29e-109	HotNet2/ReMIC
NOTCH1	5	1.54e-78	HotNet2/ReMIC
PIK3CA	6	1.54e-78	HotNet2/ReMIC
CASP8	11	6.94e-37	
NSD1	12	2.24e-31	ReMIC
LAMA2	23	2.34e-21	HotNet2/ReMIC
FBXW7	36	3.44e-15	HotNet2/ReMIC
EP300	42	3.51e-14	
HRAS	43	3.51e-14	
MACF1	44	3.51e-14	HotNet2/ReMIC
NOTCH2	48	4.09e-12	HotNet2/ReMIC
HLA-A	58	3.66e-11	HotNet2
ATR	67	3.06e-10	ReMIC
KALRN	78	2.45e-09	ReMIC
NFE2L2	80	2.45e-09	HotNet2/ReMIC
EPHA2	86	1.91e-08	HotNet2
EGFR	103	1.32e-07	ReMIC
CYLD	116	2.04e-07	
MYH9	135	8.53e-07	ReMIC
TGFBR2	144	8.53e-07	
ARID2	157	4.80e-06	HotNet2/ReMIC
CTCF	160	4.80e-06	HotNet2
ATRX	184	9.47e-06	ReMIC
APC	204	2.63e-05	ReMIC
ATM	205	2.63e-05	ReMIC
HLA-B	214	2.63e-05	HotNet2
RASA1	223	2.63e-05	ReMIC
SMARCA4	225	2.63e-05	ReMIC
SPTAN1	227	2.63e-05	ReMIC
NCOR1	247	8.22e-05	
BAZ2B	266	1.29e-04	ReMIC
KDM6A	282	1.29e-04	HotNet2
TRIO	301	1.29e-04	ReMIC
FN1	324	4.63e-04	ReMIC
CHD9	347	5.76e-04	ReMIC
ARID1B	403	2.33e-03	ReMIC
CUL3	415	2.33e-03	
MEF2C	438	2.33e-03	

continued on next page

continued from previous page

PBRM1	451	2.33e-03	HotNet2
RAC1	461	2.33e-03	ReMIC
TAOK2	470	2.33e-03	
APAF1	523	8.96e-03	
PCDH18	586	8.96e-03	ReMIC
NF1	608	9.44e-03	ReMIC
CIITA	677	2.01e-02	
ARHGAP35	688	2.95e-02	HotNet2/ReMIC
BRCA1	694	2.95e-02	ReMIC
DICER1	704	2.95e-02	HotNet2
DNMT3A	707	2.95e-02	ReMIC
MTOR	753	2.95e-02	ReMIC
PABPC3	765	2.95e-02	ReMIC
WHSC1	810	2.95e-02	
B2M	826	3.58e-02	
ARFGEF2	836	4.07e-02	HotNet2
BRWD1	839	4.07e-02	
CUL1	843	4.07e-02	
HSPA8	853	4.07e-02	

(E) IntOGen genes in LAML results

Gene Symbol	Rank	q-value	also detected by
NPM1	1	5.18e-165	mCGfinder/HotNet2
DNMT3A	2	1.03e-93	mCGfinder/HotNet2/ReMIC
FLT3	3	4.11e-87	mCGfinder/ReMIC
RUNX1	4	2.22e-45	mCGfinder/HotNet2
IDH2	5	1.48e-35	mCGfinder
IDH1	6	1.99e-26	mCGfinder
TP53	7	4.08e-25	mCGfinder/HotNet2
TET2	8	8.60e-11	mCGfinder/HotNet2
PTPN11	9	5.07e-10	mCGfinder/HotNet2/ReMIC
NRAS	10	1.13e-09	mCGfinder/HotNet2
ASXL1	12	1.47e-07	mCGfinder
WT1	13	1.55e-07	mCGfinder/HotNet2
CEBPA	15	3.79e-06	mCGfinder/HotNet2
KIT	16	4.17e-06	mCGfinder/HotNet2
RAD21	17	7.77e-05	mCGfinder/HotNet2
STAG2	18	7.82e-05	mCGfinder/HotNet2/ReMIC
U2AF1	19	6.90e-04	mCGfinder/HotNet2
KRAS	23	1.45e-03	mCGfinder/HotNet2/ReMIC
PHF6	24	2.41e-03	mCGfinder
SUZ12	26	3.38e-03	mCGfinder/HotNet2
PRPF8	30	1.82e-02	mCGfinder/HotNet2

Supplementary Table S3. The CGC and IntOGen genes identified by mCGfinder but not by the other investigated methods along with their functions in literature.

Gene	Gene name	Cancer (reported by)	Function	PubMed ID
ACTB	Actin Beta	BLCA (IntOGen)	Actins are highly conserved proteins that are involved in various types of cell motility and are ubiquitously expressed in all eukaryotic cells.	23266771; 16685646; 1734024; 15940343
AKAP9	A-Kinase Anchoring Protein 9	BRCA (IntOGen)	Scaffolding protein that assembles several protein kinases and phosphatases on the centrosome and Golgi apparatus. Required to maintain the integrity of the Golgi apparatus. Recruited to the Golgi apparatus by GM130/GOLGA2 and is required for microtubule nucleation at the cis-side of the Golgi apparatusGM130/GOLGA2.	10202149; 15047863; 19242490
AKT1	AKT Serine/Threonine Kinase 1	BRCA (CGC & IntOGen)	AKT1 is one of 3 closely related serine/threonine-protein kinases (AKT1, AKT2 and AKT3) called the AKT kinase, and which regulate many processes including metabolism, proliferation, cell survival, growth and angiogenesis. This is mediated through serine and/or threonine phosphorylation of a range of downstream substrates. Over 100 substrate candidates have been reported so far, but for most of them, no isoform specificity has been reported. AKT is responsible of the regulation of glucose uptake by mediating insulin-induced translocation of the SLC2A4/GLUT4 glucose transporter to the cell surface. Phosphorylation of PTPN1 at 'Ser-50' negatively modulates its phosphatase activity preventing dephosphorylation of the insulin receptor and the attenuation of insulin signaling. Phosphorylation of TBC1D4 triggers the binding of this effector to inhibitory 14-3-3 proteins, which is required for insulin-stimulated glucose transport. AKT regulates also the storage of glucose in the form of glycogen by phosphorylating GSK3A at 'Ser-21' and GSK3B at 'Ser-9', resulting in inhibition of its kinase activity. Phosphorylation of GSK3 isoforms by AKT is also thought to be one mechanism by which cell proliferation is driven. AKT regulates also cell survival via the phosphorylation of MAP3K5 (apoptosis signal-related kinase). Phosphorylation of 'Ser-83' decreases MAP3K5 kinase activity stimulated by oxidative stress and thereby prevents apoptosis. AKT mediates insulin-stimulated protein synthesis by phosphorylating TSC2 at 'Ser-939' and 'Thr-1462', thereby activating mTORC1 signaling and leading to both phosphorylation of 4E-BP1 and in activation of RPS6KB1. AKT is involved in the phosphorylation of members of the FOXO factors (Forkhead family of transcription factors), leading to binding of 14-3-3 proteins and cytoplasmic localization. In particular, FOXO1 is phosphorylated at 'Thr-24', 'Ser-256' and 'Ser-319'. FOXO3 and FOXO4 are phosphorylated on equivalent sites. AKT has an important role in the regulation of NF-kappa-B-dependent gene transcription and positively regulates the activity of CREB1 (cyclic AMP (cAMP)-response element binding protein). The phosphorylation of CREB1 induces the binding of accessory proteins that are necessary for the transcription of pro-survival genes such as BCL2 and MCL1. AKT phosphorylates 'Ser-454' on ATP citrate lyase (ACLY), thereby potentially regulating ACLY activity and fatty acid synthesis. Activates the 3B isoform of cyclic nucleotide phosphodiesterase (PDE3B) via phosphorylation of 'Ser-273', resulting in reduced cyclic AMP levels and inhibition of lipolysis. Phosphorylates PIKFYVE on 'Ser-318', which results in increased PI3P-5 activity. The Rho GTPase-activating protein DLC1 is another substrate and its phosphorylation is implicated in the regulation cell proliferation and cell growth. AKT plays a role as key modulator of the AKT-mTOR signaling pathway controlling the tempo of the process of newborn neurons integration during adult neurogenesis, including correct neuron positioning, dendritic development and synapse formation. Signals downstream of phosphatidylinositol 3-kinase (PI3K) to mediate the effects of various growth factors such as platelet-derived growth factor (PDGF), epidermal growth factor (EGF), insulin and insulin-like growth factor I (IGF-I). AKT mediates the antiapoptotic effects of IGF-I. Essential for the SPATA13-mediated regulation of cell migration and adhesion assembly and disassembly. May be involved in the regulation of the placental development. Phosphorylates STK4/MST1 at 'Thr-120' and 'Thr-387' leading to inhibition of its: kinase activity, nuclear translocation, autophosphorylation and ability to phosphorylate FOXO3. Phosphorylates STK3/MST2 at 'Thr-117' and 'Thr-384' leading to inhibition of its: cleavage, kinase activity, autophosphorylation at Thr-180, binding to RASSF1 and nuclear translocation. Phosphorylates SRPK2 and enhances its kinase activity towards SRSF2 and ACIN1 and promotes its nuclear translocation. Phosphorylates RAF1 at 'Ser-259' and negatively regulates its activity. Phosphorylation of BAD stimulates its pro-apoptotic activity.	28339062; 27717743; 10330141; 27166089; 28287129
APAF1	Apoptotic Peptidase Activating Factor 1	HNSC (IntOGen)	Oligomeric Apaf-1 mediates the cytochrome c-dependent autocatalytic activation of pro-caspase-9 (Apaf-3), leading to the activation of caspase-3 and apoptosis. This activation requires ATP. Isoform 6 is less effective in inducing apoptosis.	10393175; 12804598
ASH1L	ASH1 Like Histone Lysine Methyltransferase	BRCA (IntOGen)	Histone methyltransferase specifically methylating 'Lys-36' of histone H3 (H3K36me).	21239497
ASXL1	Additional Sex Combs Like 1, Transcriptional Regulator	LAML (IntOGen)	Probable Polycomb group (PcG) protein involved in transcriptional regulation mediated by ligand-bound nuclear hormone receptors, such as retinoic acid receptors (RARs) and peroxisome proliferator-activated receptor gamma (PPARG). Acts as coactivator of RARA and RXRA through association with NCOA1. Acts as corepressor through recruitment of KDM1A and CBX5 to target genes in a cell-type specific manner; the function seems to involve differential recruitment of methylated histone H3 to respective promoters. Acts as corepressor for PPARG and suppresses its adipocyte differentiation-inducing activity . Non-catalytic component of the PR-DUB complex, a complex that specifically mediates deubiquitination of histone H2A monoubiquitinated at 'Lys-119' (H2AK119ub1).	16606617; 20436459
ATM	ATM Serine/Threonine Kinase	BRCA (IntOGen)	Serine/threonine protein kinase which activates checkpoint signaling upon double strand breaks (DSBs), apoptosis and genotoxic stresses such as ionizing ultraviolet A light (UVA), thereby acting as a DNA damage sensor. Recognizes the substrate consensus sequence [ST]-Q. Phosphorylates 'Ser-139' of histone variant H2AX/H2AFX at double strand breaks (DSBs), thereby regulating DNA damage response mechanism. Also plays a role in pre-B cell allelic exclusion, a process leading to expression of a single immunoglobulin heavy chain allele to enforce clonality and monospecific recognition by the B-cell antigen receptor (BCR) expressed on individual B-lymphocytes. After the introduction of DNA breaks by the RAG complex on one immunoglobulin allele, acts by mediating a repositioning of the second allele to pericentromeric heterochromatin, preventing accessibility to the RAG complex and recombination of the second allele. Also involved in signal transduction and cell cycle control. May function as a tumor suppressor. Necessary for activation of ABL1 and SAPK. Phosphorylates DYRK2, CHEK2, p53/TP53, FANCD2, NFKBIA, BRCA1, CTIP, nibrin (NBN), TERF1, RAD9 and DCLRE1C. May play a role in vesicle and/or protein transport. Could play a role in T-cell development, gonad and neurological function. Plays a role in replication-dependent histone mRNA degradation. Binds DNA ends. Phosphorylation of DYRK2 in nucleus in response to genotoxic stress prevents its MDM2-mediated ubiquitination and subsequent proteasome degradation. Phosphorylates ATF2 which stimulates its function in DNA damage response.	12556884; 14871926; 16858402; 17923702; 19965871
B2M	Beta-2-Microglobulin	HNSC (IntOGen)	Component of the class I major histocompatibility complex (MHC). Involved in the presentation of peptide antigens to the immune system. Exogenously applied M.tuberculosis EsxA or EsxA-EsxB (or EsxA expressed in host) binds B2M and decreases its export to the cell surface (total protein levels do not change), probably leading to defects in class I antigen presentation.	25356553
BRCA2	BRCA2, DNA Repair Associated	BRCA (CGC & IntOGen)	Involved in double-strand break repair and/or homologous recombination. Binds RAD51 and potentiates recombinational DNA repair by promoting assembly of RAD51 onto single-stranded DNA (ssDNA). Acts by targeting RAD51 to ssDNA over double-stranded DNA, enabling RAD51 to displace replication protein-A (RPA) from ssDNA and stabilizing RAD51-ssDNA filaments by blocking ATP hydrolysis. Part of a PALB2-scaffolded HR complex containing RAD51C and which is thought to play a role in DNA repair by HR. May participate in S phase checkpoint activation. Binds selectively to ssDNA, and to ssDNA in tailed duplexes and replication fork structures. May play a role in the extension step after strand invasion at replication-dependent DNA double-strand breaks; together with PALB2 is involved in both POLH localization at collapsed replication forks and DNA polymerization activity. In concert with NPM1, regulates centrosome duplication. Interacts with the TREX-2 complex (transcription and export complex 2) subunits PCID2 and SEM1, and is required to prevent R-loop-associated DNA damage and thus transcription-associated genomic instability. Silencing of BRCA2 promotes R-loop accumulation at actively transcribed genes in replicating and non-replicating cells, suggesting that BRCA2 mediates the control of R-loop associated genomic instability, independently of its known role in homologous recombination.	15199141; 20729858; 21084279; 24896180; 21719596
BRWD1	Bromodomain And WD Repeat Domain Containing 1	HNSC (IntOGen)	May be a transcriptional activator. May be involved in chromatin remodeling . Plays a role in the regulation of cell morphology and cytoskeletal organization. Required in the control of cell shape.	21834987
CAD	Carbamoyl-Phosphate Synthetase 2, Aspartate Transcarbamylase, And Dihydroorotase	BRCA (IntOGen)	This protein is a "fusion" protein encoding four enzymatic activities of the pyrimidine pathway (GATase, CPSase, ATCase and DHOase).	24332717
CASP8	Caspase 8	BRCA (CGC & IntOGen); HNSC (IntOGen)	Most upstream protease of the activation cascade of caspases responsible for the TNFRSF6/FAS mediated and TNFRSF1A induced cell death. Binding to the adapter molecule FADD recruits it to either receptor. The resulting aggregate called death-inducing signaling complex (DISC) performs CASP8 proteolytic activation. The active dimeric enzyme is then liberated from the DISC and free to activate downstream apoptotic proteases. Proteolytic fragments of the N-terminal propeptide (termed CAP3, CAP5 and CAP6) are likely retained in the DISC. Cleaves and activates CASP3, CASP4, CASP6, CASP7, CASP9 and CASP10. May participate in the GZMB apoptotic pathways. Cleaves ADPRT. Hydrolyzes the small-molecule substrate, Ac-Asp-Glu-Val-Asp-|-AMC. Likely target for the cowpox virus CRMA death inhibitory protein. Isoform 5, isoform 6, isoform 7 and isoform 8 lack the catalytic site and may interfere with the pro-apoptotic activity of the complex.	9006941; 23516580
CDKN1A	Cyclin Dependent Kinase Inhibitor 1A	BLCA (IntOGen)	May be involved in p53/TP53 mediated inhibition of cellular proliferation in response to DNA damage. Binds to and inhibits cyclin-dependent kinase activity, preventing phosphorylation of critical cyclin-dependent kinase substrates and blocking cell cycle progression. Functions in the nuclear localization and assembly of cyclin D-CDK4 complex and promotes its kinase activity towards RB1. At higher stoichiometric ratios, inhibits the kinase activity of the cyclin D-CDK4 complex. Inhibits DNA synthesis by DNA polymerase delta by competing with POLD3 for PCNA binding.	8242751; 9106657; 11595739
CHD3	Chromodomain Helicase DNA Binding Protein 3	BLCA (IntOGen)	Component of the histone deacetylase NuRD complex which participates in the remodeling of chromatin by deacetylating histones. Required for anchoring centrosomal pericentrin in both interphase and mitosis, for spindle organization and centrosome integrity.	9804427; 17626165
CHD4	Chromodomain Helicase DNA Binding Protein 4	BRCA (IntOGen)	Component of the histone deacetylase NuRD complex which participates in the remodeling of chromatin by deacetylating histones.	9804427; 17626165
CHEK2	Checkpoint Kinase 2	BLCA (IntOGen)	Serine/threonine-protein kinase which is required for checkpoint-mediated cell cycle arrest, activation of DNA repair and apoptosis in response to the presence of DNA double-strand breaks. May also negatively regulate cell cycle progression during unperturbed cell cycles. Following activation, phosphorylates numerous effectors preferentially at the consensus sequence [L-X-R-X-X-S/T]. Regulates cell cycle checkpoint arrest through phosphorylation of CDC25A, CDC25B and CDC25C, inhibiting their activity. Inhibition of CDC25 phosphatase activity leads to increased inhibitory tyrosine phosphorylation of CDK-cyclin complexes and blocks cell cycle progression. May also phosphorylate NEK6 which is involved in G2/M cell cycle arrest. Regulates DNA repair through phosphorylation of BRCA2, enhancing the association of RAD51 with chromatin which promotes DNA repair by homologous recombination. Also stimulates the transcription of genes involved in DNA repair (including BRCA2) through the phosphorylation and activation of the transcription factor FOXM1. Regulates apoptosis through the phosphorylation of p53/TP53, MDM4 and PML. Phosphorylation of p53/TP53 at 'Ser-20' by CHEK2 may alleviate inhibition by MDM2, leading to accumulation of active p53/TP53. Phosphorylation of MDM4 may also reduce degradation of p53/TP53. Also controls the transcription of pro-apoptotic genes through phosphorylation of the transcription factor E2F1. Tumor suppressor, it may also have a DNA damage-independent function in mitotic spindle assembly by phosphorylating BRCA1. Its absence may be a cause of the chromosomal instability observed in some cancer cells. Promotes the CCAR2-SIRT1 association and is required for CCAR2-mediated SIRT1 inhibition.	12402044; 17715138; 18644861; 18317453; 25361978;
CIITA	Class II Major Histocompatibility Complex Transactivator	HNSC (IntOGen)	Essential for transcriptional activity of the HLA class II promoter; activation is via the proximal promoter. No DNA binding of in vitro translated CIITA was detected. May act in a coactivator-like fashion through protein-protein interactions by contacting factors binding to the proximal MHC class II promoter, to elements of the transcription machinery, or both. Alternatively it may activate HLA class II transcription by modifying proteins that bind to the MHC class II promoter. Also mediates enhanced MHC class I transcription; the promoter element requirements for CIITA-mediated transcription are distinct from those of constitutive MHC class I transcription, and CIITA can functionally replace TAF1 at these genes. Exhibits intrinsic GTP-stimulated acetyltransferase activity. Exhibits serine/threonine protein kinase activity: can phosphorylate the TFIID component TAF7, the RAP74 subunit of the general transcription factor TFIIF, histone H2B at 'Ser-37' and other histones (in vitro).	11172716; 17493635; 16600381; 24036077
CLSPN	Claspin	BLCA (IntOGen)	Required for checkpoint mediated cell cycle arrest in response to inhibition of DNA replication or to DNA damage induced by both ionizing and UV irradiation. Adapter protein which binds to BRCA1 and the checkpoint kinase CHEK1 and facilitates the ATR-dependent phosphorylation of both proteins. Can also bind specifically to branched DNA structures and may associate with S-phase chromatin following formation of the pre-replication complex (pre-RC). This may indicate a role for this protein as a sensor which monitors the integrity of DNA replication forks.	12766152; 15190204; 15226314; 15096610; 15707391
CLTC	Clathrin Heavy Chain	BLCA (IntOGen)	Clathrin is the major protein of the polyhedral coat of coated pits and vesicles. Two different adapter protein complexes link the clathrin lattice either to the plasma membrane or to the trans-Golgi network. Acts as component of the TACC3/ch-TOG/clathrin complex proposed to contribute to stabilization of kinetochore fibers of the mitotic spindle by acting as inter-microtubule bridge. The TACC3/ch-TOG/clathrin complex is required for the maintenance of kinetochore fiber tension. Plays a role in early autophagosome formation.	15858577; 16968737; 20639872; 21297582; 23532825
CTCF	CCCTC-Binding Factor	BRCA (CGC & IntOGen)	Chromatin binding factor that binds to DNA sequence specific sites. Involved in transcriptional regulation by binding to chromatin insulators and preventing interaction between promoter and nearby enhancers and silencers. Acts as transcriptional repressor binding to promoters of vertebrate MYC gene and BAG1 gene. Also binds to the PLK and PIM1 promoters. Acts as a transcriptional activator of APP. Regulates APOA1/C3/A4/A5 gene cluster and controls MHC class II gene expression. Plays an essential role in oocyte and preimplantation embryo development by activating or repressing transcription. Seems to act as tumor suppressor. Plays a critical role in the epigenetic regulation. Participates in the allele-specific gene expression at the imprinted IGF2/H19 gene locus. On the maternal allele, binding within the H19 imprinting control region (ICR) mediates maternally inherited higher-order chromatin conformation to restrict enhancer access to IGF2. Plays a critical role in gene silencing over considerable distances in the genome. Preferentially interacts with unmethylated DNA, preventing spreading of CpG methylation and maintaining methylation-free zones. Inversely, binding to target sites is prevented by CpG methylation. Plays a important role in chromatin remodeling. Can dimerize when it is bound to different DNA sequences, mediating long-range chromatin looping. Mediates interchromosomal association between IGF2/H19 and WSB1/NF1 and may direct distant DNA segments to a common transcription factory. Causes local loss of histone acetylation and gain of histone methylation in the beta-globin locus, without affecting transcription. When bound to chromatin, it provides an anchor point for nucleosomes positioning. Seems to be essential for homologous X-chromosome pairing. May participate with Tsix in establishing a regulatable epigenetic switch for X chromosome inactivation. May play a role in preventing the propagation of stable methylation at the escape genes from X- inactivation. Involved in sister chromatid cohesion. Associates with both centromeres and chromosomal arms during metaphase and required for cohesin localization to CTCF sites. Regulates asynchronous replication of IGF2/H19. Plays a role in the recruitment of CENPE to the pericentromeric/centromeric regions of the chromosome during mitosis.	8649389; 9591631; 16815976; 17827499; 18654629
CUL1	Cullin 1	HNSC (IntOGen)	Core component of multiple cullin-RING-based SCF (SKP1-CUL1-F-box protein) E3 ubiquitin-protein ligase complexes, which mediate the ubiquitination of proteins involved in cell cycle progression, signal transduction and transcription. SCF complexes and ARIH1 collaborate in tandem to mediate ubiquitination of target proteins. In the SCF complex, serves as a rigid scaffold that organizes the SKP1-F-box protein and RBX1 subunits. May contribute to catalysis through positioning of the substrate and the ubiquitin-conjugating enzyme. The E3 ubiquitin-protein ligase activity of the complex is dependent on the neddylation of the cullin subunit and exchange of the substrate recognition component is mediated by TIP120A/CAND1. The functional specificity of the SCF complex depends on the F-box protein as substrate recognition component. SCF(BTRC) and SCF(FBXW11) direct ubiquitination of CTNNB1 and participate in Wnt signaling. SCF(FBXW11) directs ubiquitination of phosphorylated NFKBIA. SCF(BTRC) directs ubiquitination of NFKBIB, NFKBIE, ATF4, SMAD3, SMAD4, CDC25A, FBXO5 and probably NFKB2. SCF(BTRC) and/or SCF(FBXW11) direct ubiquitination of CEP68. SCF(SKP2) directs ubiquitination of phosphorylated CDKN1B/p27kip and is involved in regulation of G1/S transition. SCF(SKP2) directs ubiquitination of ORC1, CDT1, RBL2, ELF4, CDKN1A, RAG2, FOXO1A, and probably MYC and TAL1. SCF(FBXW7) directs ubiquitination of cyclin E, NOTCH1 released notch intracellular domain (NICD), and probably PSEN1. SCF(FBXW2) directs ubiquitination of GCM1. SCF(FBXO32) directs ubiquitination of MYOD1. SCF(FBXO7) directs ubiquitination of BIRC2 and DLGAP5. SCF(FBXO33) directs ubiquitination of YBX1. SCF(FBXO1) directs ubiquitination of BCL6 and DTL but does not seem to direct ubiquitination of TP53. SCF(BTRC) mediates the ubiquitination of NFKBIA at 'Lys-21' and 'Lys-22'; the degradation frees the associated NFKB1-RELA dimer to translocate into the nucleus and to activate transcription. SCF(CCNF) directs ubiquitination of CCP110. SCF(FBXL3) and SCF(FBXL21) direct ubiquitination of CRY1 and CRY2. SCF(FBXO9) directs ubiquitination of TTI1 and TELO2. SCF(FBXO10) directs ubiquitination of BCL2.	9663463; 15531760; 19679664; 23431138; 25704143
CUL3	Cullin 3	HNSC (IntOGen)	Core component of multiple cullin-RING-based BCR (BTB-CUL3-RBX1) E3 ubiquitin-protein ligase complexes which mediate the ubiquitination and subsequent proteasomal degradation of target proteins. BCR complexes and ARIH1 collaborate in tandem to mediate ubiquitination of target proteins. As a scaffold protein may contribute to catalysis through positioning of the substrate and the ubiquitin-conjugating enzyme. The E3 ubiquitin-protein ligase activity of the complex is dependent on the neddylation of the cullin subunit and is inhibited by the association of the deneddylated cullin subunit with TIP120A/CAND1. The functional specificity of the BCR complex depends on the BTB domain-containing protein as the substrate recognition component. BCR(KLHL42) is involved in ubiquitination of KATNA1. BCR(SPOP) is involved in ubiquitination of BMI1/PCGF4, BRMS1, H2AFY and DAXX, GLI2 and GLI3. Can also form a cullin-RING-based BCR (BTB-CUL3-RBX1) E3 ubiquitin-protein ligase complex containing homodimeric SPOPL or the heterodimer formed by SPOP and SPOPL; these complexes have lower ubiquitin ligase activity. BCR(KLHL9-KLHL13) controls the dynamic behavior of AURKB on mitotic chromosomes and thereby coordinates faithful mitotic progression and completion of cytokinesis. BCR(KLHL12) is involved in ER-Golgi transport by regulating the size of COPII coats, thereby playing a key role in collagen export, which is required for embryonic stem (ES) cells division: BCR(KLHL12) acts by mediating monoubiquitination of SEC31 (SEC31A or SEC31B). BCR(KLHL3) acts as a regulator of ion transport in the distal nephron; by mediating ubiquitination of WNK4. The BCR(KLHL20) E3 ubiquitin ligase complex is involved in interferon response and anterograde Golgi to endosome transport: it mediates both ubiquitination leading to degradation and 'Lys-33'-linked ubiquitination. The BCR(KLHL21) E3 ubiquitin ligase complex regulates localization of the chromosomal passenger complex (CPC) from chromosomes to the spindle midzone in anaphase and mediates the ubiquitination of AURKB. The BCR(KLHL22) ubiquitin ligase complex mediates monoubiquitination of PLK1, leading to PLK1 dissociation from phosphoreceptor proteins and subsequent removal from kinetochores, allowing silencing of the spindle assembly checkpoint (SAC) and chromosome segregation. The BCR(KLHL25) ubiquitin ligase complex is involved in translational homeostasis by mediating ubiquitination and subsequent degradation of hypophosphorylated EIF4EBP1 (4E-BP1). Involved in ubiquitination of cyclin E and of cyclin D1 (in vitro) thus involved in regulation of G1/S transition. Involved in the ubiquitination of KEAP1, ENC1 and KLHL41. In concert with ATF2 and RBX1, promotes degradation of KAT5 thereby attenuating its ability to acetylate and activate ATM. The BCR(KCTD17) E3 ubiquitin ligase complex mediates ubiquitination and degradation of TCHP, a down-regulator of cilium assembly, thereby inducing ciliogenesis.	10500095; 11311237; 15983046; 18397884; 20389280; 25270598;
CYLD	CYLD Lysine 63 Deubiquitinase	HNSC (IntOGen)	Deubiquitinase that specifically cleaves 'Lys-63'-linked polyubiquitin chains. Has endodeubiquitinase activity. Plays an important role in the regulation of pathways leading to NF-kappa-B activation. Contributes to the regulation of cell survival, proliferation and differentiation via its effects on NF-kappa-B activation. Negative regulator of Wnt signaling. Inhibits HDAC6 and thereby promotes acetylation of alpha-tubulin and stabilization of microtubules. Plays a role in the regulation of microtubule dynamics, and thereby contributes to the regulation of cell proliferation, cell polarization, cell migration, and angiogenesis. Required for normal cell cycle progress and normal cytokinesis. Inhibits nuclear translocation of NF-kappa-B. Plays a role in the regulation of inflammation and the innate immune response, via its effects on NF-kappa-B activation. Dispensable for the maturation of intrathymic natural killer cells, but required for the continued survival of immature natural killer cells. Negatively regulates TNFRSF11A signaling and osteoclastogenesis . Involved in the regulation of ciliogenesis, allowing ciliary basal bodies to migrate and dock to the plasma membrane; this process does not depend on NF-kappa-B activation . Also able to remove linear ('Met-1'-linked) polyubiquitin chains to regulate innate immunity: recruited to the LUBAC complex and, together with OTULIN, restricts linear polyubiquitin formation on RIPK2 in response to NOD2 stimulation.	12917689; 14676304; 18636086; 18222923; 20227366; 26670046;
EP300	E1A Binding Protein P300	HNSC (IntOGen)	Functions as histone acetyltransferase and regulates transcription via chromatin remodeling. Acetylates all four core histones in nucleosomes. Histone acetylation gives an epigenetic tag for transcriptional activation. Mediates cAMP-gene regulation by binding specifically to phosphorylated CREB protein. Mediates acetylation of histone H3 at 'Lys-122' (H3K122ac), a modification that localizes at the surface of the histone octamer and stimulates transcription, possibly by promoting nucleosome instability. Mediates acetylation of histone H3 at 'Lys-27' (H3K27ac). Also functions as acetyltransferase for nonhistone targets. Acetylates 'Lys-131' of ALX1 and acts as its coactivator. Acetylates SIRT2 and is proposed to indirectly increase the transcriptional activity of TP53 through acetylation and subsequent attenuation of SIRT2 deacetylase function. Acetylates HDAC1 leading to its inactivation and modulation of transcription. Acts as a TFAP2A-mediated transcriptional coactivator in presence of CITED2. Plays a role as a coactivator of NEUROD1-dependent transcription of the secretin and p21 genes and controls terminal differentiation of cells in the intestinal epithelium. Promotes cardiac myocyte enlargement. Can also mediate transcriptional repression. Binds to and may be involved in the transforming capacity of the adenovirus E1A protein. In case of HIV-1 infection, it is recruited by the viral protein Tat. Regulates Tat's transactivating activity and may help inducing chromatin remodeling of proviral genes. Acetylates FOXO1 and enhances its transcriptional activity. Acetylates BCL6 wich disrupts its ability to recruit histone deacetylases and hinders its transcriptional repressor activity. Participates in CLOCK or NPAS2-regulated rhythmic gene transcription; exhibits a circadian association with CLOCK or NPAS2, correlating with increase in PER1/2 mRNA and histone H3 acetylation on the PER1/2 promoter. Acetylates MTA1 at 'Lys-626' which is essential for its transcriptional coactivator activity. Acetylates XBP1 isoform 2; acetylation increases protein stability of XBP1 isoform 2 and enhances its transcriptional activity. Acetylates PCNA; acetylation promotes removal of chromatin-bound PCNA and its degradation during nucleotide excision repair (NER). Acetylates MEF2D.	11701890; 8945521; 14645221; 15890677; 18722353; 20955178; 23934153
ERCC2	ERCC Excision Repair 2, TFIIH Core Complex Helicase Subunit	BLCA (IntOGen)	ATP-dependent 5'-3' DNA helicase, component of the core-TFIIH basal transcription factor. Involved in nucleotide excision repair (NER) of DNA by opening DNA around the damage, and in RNA transcription by RNA polymerase II by anchoring the CDK-activating kinase (CAK) complex, composed of CDK7, cyclin H and MAT1, to the core-TFIIH complex. Involved in the regulation of vitamin-D receptor activity. As part of the mitotic spindle-associated MMXD complex it plays a role in chromosome segregation. Might have a role in aging process and could play a causative role in the generation of skin cancers.	8413672; 10024882; 15494306; 20797633
HCFC1	Host Cell Factor C1	BRCA (IntOGen)	Involved in control of the cell cycle. Also antagonizes transactivation by ZBTB17 and GABP2; represses ZBTB17 activation of the p15(INK4b) promoter and inhibits its ability to recruit p300. Coactivator for EGR2 and GABP2. Tethers the chromatin modifying Set1/Ash2 histone H3 'Lys-4' methyltransferase (H3K4me) and Sin3 histone deacetylase (HDAC) complexes (involved in the activation and repression of transcription, respectively) together. Component of a THAP1/THAP3-HCFC1-OGT complex that is required for the regulation of the transcriptional activity of RRM1. As part of the NSL complex it may be involved in acetylation of nucleosomal histone H4 on several lysine residues. In case of human herpes simplex virus (HSV) infection, HCFC1 forms a multiprotein-DNA complex with the viral transactivator protein VP16 and POU2F1 thereby enabling the transcription of the viral immediate early genes.	10920196; 9990006; 10675337; 12244100; 15190068; 20200153
HRAS	HRas Proto-Oncogene, GTPase	HNSC (IntOGen)	Ras proteins bind GDP/GTP and possess intrinsic GTPase activity.	14500341; 9020151; 12740440
HSP90AA1	Heat Shock Protein 90 Alpha Family Class A Member 1	BLCA (IntOGen)	Molecular chaperone that promotes the maturation, structural maintenance and proper regulation of specific target proteins involved for instance in cell cycle control and signal transduction. Undergoes a functional cycle that is linked to its ATPase activity which is essential for its chaperone activity. This cycle probably induces conformational changes in the client proteins, thereby causing their activation. Interacts dynamically with various co-chaperones that modulate its substrate recognition, ATPase cycle and chaperone function. Engages with a range of client protein classes via its interaction with various co-chaperone proteins or complexes, that act as adapters, simultaneously able to interact with the specific client and the central chaperone itself. Recruitment of ATP and co-chaperone followed by client protein forms a functional chaperone. After the completion of the chaperoning process, properly folded client protein and co-chaperone leave HSP90 in an ADP-bound partially open conformation and finally, ADP is released from HSP90 which acquires an open conformation for the next cycle. Apart from its chaperone activity, it also plays a role in the regulation of the transcription machinery. HSP90 and its co-chaperones modulate transcription at least at three different levels. In the first place, they alter the steady-state levels of certain transcription factors in response to various physiological cues. Second, they modulate the activity of certain epigenetic modifiers, such as histone deacetylases or DNA methyl transferases, and thereby respond to the change in the environment. Third, they participate in the eviction of histones from the promoter region of certain genes and thereby turn on gene expression. Binds bacterial lipopolysaccharide (LPS) and mediates LPS-induced inflammatory response, including TNF secretion by monocytes. Antagonizes STUB1-mediated inhibition of TGF-beta signaling via inhibition of STUB1-mediated SMAD3 ubiquitination and degradation.	25973397; 27295069; 26991466; 15937123; 11274138; 27353360
HSP90AB1	Heat Shock Protein 90 Alpha Family Class B Member 1	BLCA (IntOGen)	Molecular chaperone that promotes the maturation, structural maintenance and proper regulation of specific target proteins involved for instance in cell cycle control and signal transduction. Undergoes a functional cycle that is linked to its ATPase activity. This cycle probably induces conformational changes in the client proteins, thereby causing their activation. Interacts dynamically with various co-chaperones that modulate its substrate recognition, ATPase cycle and chaperone function. Engages with a range of client protein classes via its interaction with various co-chaperone proteins or complexes, that act as adapters, simultaneously able to interact with the specific client and the central chaperone itself. Recruitment of ATP and co-chaperone followed by client protein forms a functional chaperone. After the completion of the chaperoning process, properly folded client protein and co-chaperone leave HSP90 in an ADP-bound partially open conformation and finally, ADP is released from HSP90 which acquires an open conformation for the next cycle. Apart from its chaperone activity, it also plays a role in the regulation of the transcription machinery. HSP90 and its co-chaperones modulate transcription at least at three different levels. In the first place, they alter the steady-state levels of certain transcription factors in response to various physiological cues. Second, they modulate the activity of certain epigenetic modifiers, such as histone deacetylases or DNA methyl transferases, and thereby respond to the change in the environment. Third, they participate in the eviction of histones from the promoter region of certain genes and thereby turn on gene expression. Antagonizes STUB1-mediated inhibition of TGF-beta signaling via inhibition of STUB1-mediated SMAD3 ubiquitination and degradation.	25973397; 27295069; 26991466; 16478993; 19696785; 24613385
HSPA8	Heat Shock Protein Family A (Hsp70) Member 8	HNSC (IntOGen)	Molecular chaperone implicated in a wide variety of cellular processes, including protection of the proteome from stress, folding and transport of newly synthesized polypeptides, activation of proteolysis of misfolded proteins and the formation and dissociation of protein complexes. Plays a pivotal role in the protein quality control system, ensuring the correct folding of proteins, the re-folding of misfolded proteins and controlling the targeting of proteins for subsequent degradation. This is achieved through cycles of ATP binding, ATP hydrolysis and ADP release, mediated by co-chaperones. The co-chaperones have been shown to not only regulate different steps of the ATPase cycle of HSP70, but they also have an individual specificity such that one co-chaperone may promote folding of a substrate while another may promote degradation. The affinity of HSP70 for polypeptides is regulated by its nucleotide bound state. In the ATP-bound form, it has a low affinity for substrate proteins. However, upon hydrolysis of the ATP to ADP, it undergoes a conformational change that increases its affinity for substrate proteins. HSP70 goes through repeated cycles of ATP hydrolysis and nucleotide exchange, which permits cycles of substrate binding and release. The HSP70-associated co-chaperones are of three types: J-domain co-chaperones HSP40s (stimulate ATPase hydrolysis by HSP70), the nucleotide exchange factors (NEF) such as BAG1/2/3 (facilitate conversion of HSP70 from the ADP-bound to the ATP-bound state thereby promoting substrate release), and the TPR domain chaperones such as HOPX and STUB1. Acts as a repressor of transcriptional activation. Inhibits the transcriptional coactivator activity of CITED1 on Smad-mediated transcription. Component of the PRP19-CDC5L complex that forms an integral part of the spliceosome and is required for activating pre-mRNA splicing. May have a scaffolding role in the spliceosome assembly as it contacts all other components of the core complex. Binds bacterial lipopolysaccharide (LPS) and mediates LPS-induced inflammatory response, including TNF secretion by monocytes. Participates in the ER-associated degradation (ERAD) quality control pathway in conjunction with J domain-containing co-chaperones and the E3 ligase STUB1.	24121476; 26865365; 10722728; 11276205; 23990462; 24318877; 27474739
IDH1	Isocitrate Dehydrogenase (NADP(+)) 1, Cytosolic	LAML (IntOGen)	IDH1 (Isocitrate Dehydrogenase (NADP(+)) 1, Cytosolic) is a Protein Coding gene. Diseases associated with IDH1 include Glioma Susceptibility 1 and Metaphyseal Enchondromatosis With D-2-Hydroxyglutaric Aciduria. Among its related pathways are Metabolism and Citrate cycle (TCA cycle). GO annotations related to this gene include protein homodimerization activity and magnesium ion binding. An important paralog of this gene is IDH2.	26678339; 26338964; 25732040; 26508204; 22025298
KRAS	KRAS Proto-Oncogene, GTPase	BRCA (IntOGen)	Ras proteins bind GDP/GTP and possess intrinsic GTPase activity. Plays an important role in the regulation of cell proliferation. Plays a role in promoting oncogenic events by inducing transcriptional silencing of tumor suppressor genes (TSGs) in colorectal cancer (CRC) cells in a ZNF304-dependent manner.	22711838; 23698361; 24623306
LZTR1	Leucine Zipper Like Transcription Regulator 1	GBM (CGC)	Probable transcriptional regulator that may play a crucial role in embryogenesis.	16356934
MAP3K1	Mitogen-Activated Protein Kinase Kinase Kinase 1	BRCA (CGC & IntOGen); BLCA (IntOGen)	Component of a protein kinase signal transduction cascade. Activates the ERK and JNK kinase pathways by phosphorylation of MAP2K1 and MAP2K4. Activates CHUK and IKBKB, the central protein kinases of the NF-kappa-B pathway.	9808624
MAP3K13	Mitogen-Activated Protein Kinase Kinase Kinase 13	BRCA (CGC)	Activates the JUN N-terminal pathway through activation of the MAP kinase kinase MAP2K7. Acts synergistically with PRDX3 to regulate the activation of NF-kappa-B in the cytosol. This activation is kinase-dependent and involves activating the IKK complex, the IKBKB-containing complex that phosphorylates inhibitors of NF-kappa-B.	9353328; 11726277; 12492477
MECOM	MDS1 And EVI1 Complex Locus	BLCA (IntOGen)	Functions as a transcriptional regulator binding to DNA sequences in the promoter region of target genes and regulating positively or negatively their expression. Oncogene which plays a role in development, cell proliferation and differentiation. May also play a role in apoptosis through regulation of the JNK and TGF-beta signaling. Involved in hematopoiesis.	9665135; 10856240; 11568182; 15897867; 16462766; 19767769
MEF2C	Myocyte Enhancer Factor 2C	HNSC (IntOGen)	Transcription activator which binds specifically to the MEF2 element present in the regulatory regions of many muscle-specific genes. Controls cardiac morphogenesis and myogenesis, and is also involved in vascular development. Plays an essential role in hippocampal-dependent learning and memory by suppressing the number of excitatory synapses and thus regulating basal and evoked synaptic transmission. Crucial for normal neuronal development, distribution, and electrical activity in the neocortex. Necessary for proper development of megakaryocytes and platelets and for bone marrow B-lymphopoiesis. Required for B-cell survival and proliferation in response to BCR stimulation, efficient IgG1 antibody responses to T-cell-dependent antigens and for normal induction of germinal center B-cells. May also be involved in neurogenesis and in the development of cortical architecture . Isoform 3 and isoform 4, which lack the repressor domain, are more active than isoform 1 and isoform 2.	9384584; 9069290; 11904443; 15340086; 15834131; 15831463
MGA	MGA, MAX Dimerization Protein	BRCA (IntOGen)	Functions as a dual-specificity transcription factor, regulating the expression of both MAX-network and T-box family target genes. Functions as a repressor or an activator. Binds to 5'-AATTTCACACCTAGGTGTGAAATT-3' core sequence and seems to regulate MYC-MAX target genes. Suppresses transcriptional activation by MYC and inhibits MYC-dependent cell transformation. Function activated by heterodimerization with MAX. This heterodimerization serves the dual function of both generating an E-box-binding heterodimer and simultaneously blocking interaction of a corepressor .	10601024; 24362264; 11031250
MLH1	MutL Homolog 1	BLCA (IntOGen)	Heterodimerizes with PMS2 to form MutL alpha, a component of the post-replicative DNA mismatch repair system (MMR). DNA repair is initiated by MutS alpha (MSH2-MSH6) or MutS beta (MSH2-MSH6) binding to a dsDNA mismatch, then MutL alpha is recruited to the heteroduplex. Assembly of the MutL-MutS-heteroduplex ternary complex in presence of RFC and PCNA is sufficient to activate endonuclease activity of PMS2. It introduces single-strand breaks near the mismatch and thus generates new entry points for the exonuclease EXO1 to degrade the strand containing the mismatch. DNA methylation would prevent cleavage and therefore assure that only the newly mutated DNA strand is going to be corrected. MutL alpha (MLH1-PMS2) interacts physically with the clamp loader subunits of DNA polymerase III, suggesting that it may play a role to recruit the DNA polymerase III to the site of the MMR. Also implicated in DNA damage signaling, a process which induces cell cycle arrest and can lead to apoptosis in case of major DNA damages. Heterodimerizes with MLH3 to form MutL gamma which plays a role in meiosis.	16873062; 18206974; 20020535; 21120944; 9311737
MLL	Myeloid/Lymphoid or Mixed-Lineage Leukemia	BRCA (IntOGen)	Histone methyltransferase that plays an essential role in early development and hematopoiesis. Catalytic subunit of the MLL1/MLL complex, a multiprotein complex that mediates both methylation of 'Lys-4' of histone H3 (H3K4me) complex and acetylation of 'Lys-16' of histone H4 (H4K16ac). In the MLL1/MLL complex, it specifically mediates H3K4me, a specific tag for epigenetic transcriptional activation. Has weak methyltransferase activity by itself, and requires other component of the MLL1/MLL complex to obtain full methyltransferase activity. Has no activity toward histone H3 phosphorylated on 'Thr-3', less activity toward H3 dimethylated on 'Arg-8' or 'Lys-9', while it has higher activity toward H3 acetylated on 'Lys-9'. Required for transcriptional activation of HOXA9. Promotes PPP1R15A-induced apoptosis. Plays a critical role in the control of circadian gene expression and is essential for the transcriptional activation mediated by the CLOCK-ARNTL/BMAL1 heterodimer. Establishes a permissive chromatin state for circadian transcription by mediating a rhythmic methylation of 'Lys-4' of histone H3 (H3K4me) and this histone modification directs the circadian acetylation at H3K9 and H3K14 allowing the recruitment of CLOCK-ARNTL/BMAL1 to chromatin .	10490642; 12453419; 15960975; 19556245
MTOR	Mechanistic Target Of Rapamycin	BRCA (IntOGen)	Serine/threonine protein kinase which is a central regulator of cellular metabolism, growth and survival in response to hormones, growth factors, nutrients, energy and stress signals. MTOR directly or indirectly regulates the phosphorylation of at least 800 proteins. Functions as part of 2 structurally and functionally distinct signaling complexes mTORC1 and mTORC2 (mTOR complex 1 and 2). Activated mTORC1 up-regulates protein synthesis by phosphorylating key regulators of mRNA translation and ribosome synthesis. This includes phosphorylation of EIF4EBP1 and release of its inhibition toward the elongation initiation factor 4E (eiF4E). Moreover, phosphorylates and activates RPS6KB1 and RPS6KB2 that promote protein synthesis by modulating the activity of their downstream targets including ribosomal protein S6, eukaryotic translation initiation factor EIF4B, and the inhibitor of translation initiation PDCD4. Stimulates the pyrimidine biosynthesis pathway, both by acute regulation through RPS6KB1-mediated phosphorylation of the biosynthetic enzyme CAD, and delayed regulation, through transcriptional enhancement of the pentose phosphate pathway which produces 5-phosphoribosyl-1-pyrophosphate (PRPP), an allosteric activator of CAD at a later step in synthesis, this function is dependent on the mTORC1 complex. Regulates ribosome synthesis by activating RNA polymerase III-dependent transcription through phosphorylation and inhibition of MAF1 an RNA polymerase III-repressor. In parallel to protein synthesis, also regulates lipid synthesis through SREBF1/SREBP1 and LPIN1. To maintain energy homeostasis mTORC1 may also regulate mitochondrial biogenesis through regulation of PPARGC1A. mTORC1 also negatively regulates autophagy through phosphorylation of ULK1. Under nutrient sufficiency, phosphorylates ULK1 at 'Ser-758', disrupting the interaction with AMPK and preventing activation of ULK1. Also prevents autophagy through phosphorylation of the autophagy inhibitor DAP. mTORC1 exerts a feedback control on upstream growth factor signaling that includes phosphorylation and activation of GRB10 a INSR-dependent signaling suppressor. Among other potential targets mTORC1 may phosphorylate CLIP1 and regulate microtubules. As part of the mTORC2 complex MTOR may regulate other cellular processes including survival and organization of the cytoskeleton. Plays a critical role in the phosphorylation at 'Ser-473' of AKT1, a pro-survival effector of phosphoinositide 3-kinase, facilitating its activation by PDK1. mTORC2 may regulate the actin cytoskeleton, through phosphorylation of PRKCA, PXN and activation of the Rho-type guanine nucleotide exchange factors RHOA and RAC1A or RAC1B. mTORC2 also regulates the phosphorylation of SGK1 at 'Ser-422'. Regulates osteoclastogensis by adjusting the expression of CEBPB isoforms .	12150925; 12231510; 12718876; 15467718; 20516213; 23429703
MYB	MYB Proto-Oncogene, Transcription Factor	BRCA (IntOGen)	Transcriptional activator; DNA-binding protein that specifically recognize the sequence 5'-YAAC[GT]G-3'. Plays an important role in the control of proliferation and differentiation of hematopoietic progenitor cells.	19036881; 24860186; 18585056; 15691758
MYH10	Myosin Heavy Chain 10	BLCA (IntOGen)	Cellular myosin that appears to play a role in cytokinesis, cell shape, and specialized functions such as secretion and capping. Involved with LARP6 in the stabilization of type I collagen mRNAs for CO1A1 and CO1A2. During cell spreading, plays an important role in cytoskeleton reorganization, focal contacts formation (in the central part but not the margins of spreading cells), and lamellipodial extension; this function is mechanically antagonized by MYH9.	20603131; 20052411
MYH9	Myosin Heavy Chain 9	BRCA (IntOGen)	Cellular myosin that appears to play a role in cytokinesis, cell shape, and specialized functions such as secretion and capping. During cell spreading, plays an important role in cytoskeleton reorganization, focal contacts formation (in the margins but not the central part of spreading cells), and lamellipodial retraction; this function is mechanically antagonized by MYH10.	20052411
NAP1L1	Nucleosome Assembly Protein 1 Like 1	BLCA (IntOGen)	May be involved in modulating chromatin formation and contribute to regulation of cell proliferation.	25071868
NCOR1	Nuclear Receptor Corepressor 1	BRCA (CGC & IntOGen); HNSC (IntOGen)	Mediates transcriptional repression by certain nuclear receptors. Part of a complex which promotes histone deacetylation and the formation of repressive chromatin structures which may impede the access of basal transcription factors. Participates in the transcriptional repressor activity produced by BCL6.	14527417
NUP98	Nucleoporin 98	BLCA (IntOGen)	Plays a role in the nuclear pore complex (NPC) assembly and/or maintenance. Nup98 and Nup96 are involved in the bidirectional transport across the NPC. May anchor NUP153 and TPR to the NPC.	15229283
PHF6	PHD Finger Protein 6	LAML (IntOGen)	Transcriptional regulator that associates with ribosomal RNA promoters and suppresses ribosomal RNA (rRNA) transcription.	23229552
PIK3CB	Phosphatidylinositol-4,5-Bisphosphate 3-Kinase Catalytic Subunit Beta	BRCA (IntOGen)	Phosphoinositide-3-kinase (PI3K) that phosphorylates PtdIns (Phosphatidylinositol), PtdIns4P (Phosphatidylinositol 4-phosphate) and PtdIns(4,5)P2 (Phosphatidylinositol 4,5-bisphosphate) to generate phosphatidylinositol 3,4,5-trisphosphate (PIP3). PIP3 plays a key role by recruiting PH domain-containing proteins to the membrane, including AKT1 and PDPK1, activating signaling cascades involved in cell growth, survival, proliferation, motility and morphology. Involved in the activation of AKT1 upon stimulation by G-protein coupled receptors (GPCRs) ligands such as CXCL12, sphingosine 1-phosphate, and lysophosphatidic acid. May also act downstream receptor tyrosine kinases. Required in different signaling pathways for stable platelet adhesion and aggregation. Plays a role in platelet activation signaling triggered by GPCRs, alpha-IIb/beta-3 integrins (ITGA2B/ ITGB3) and ITAM (immunoreceptor tyrosine-based activation motif)-bearing receptors such as GP6. Regulates the strength of adhesion of ITGA2B/ ITGB3 activated receptors necessary for the cellular transmission of contractile forces. Required for platelet aggregation induced by F2 (thrombin) and thromboxane A2 (TXA2). Has a role in cell survival. May have a role in cell migration. Involved in the early stage of autophagosome formation. Modulates the intracellular level of PtdIns3P (Phosphatidylinositol 3-phosphate) and activates PIK3C3 kinase activity. May act as a scaffold, independently of its lipid kinase activity to positively regulate autophagy. May have a role in insulin signaling as scaffolding protein in which the lipid kinase activity is not required. May have a kinase-independent function in regulating cell proliferation and in clathrin-mediated endocytosis. Mediator of oncogenic signal in cell lines lacking PTEN. The lipid kinase activity is necessary for its role in oncogenic transformation. Required for the growth of ERBB2 and RAS driven tumors.	18594509; 18755892; 21030680; 21383062
PTEN	Phosphatase And Tensin Homolog	BLCA (IntOGen)	Tumor suppressor. Acts as a dual-specificity protein phosphatase, dephosphorylating tyrosine-, serine- and threonine-phosphorylated proteins. Also acts as a lipid phosphatase, removing the phosphate in the D3 position of the inositol ring from phosphatidylinositol 3,4,5-trisphosphate, phosphatidylinositol 3,4-diphosphate, phosphatidylinositol 3-phosphate and inositol 1,3,4,5-tetrakisphosphate with order of substrate preference in vitro PtdIns(3,4,5)P3 > PtdIns(3,4)P2 > PtdIns3P > Ins(1,3,4,5)P4. The lipid phosphatase activity is critical for its tumor suppressor function. Antagonizes the PI3K-AKT/PKB signaling pathway by dephosphorylating phosphoinositides and thereby modulating cell cycle progression and cell survival. The unphosphorylated form cooperates with AIP1 to suppress AKT1 activation. Dephosphorylates tyrosine-phosphorylated focal adhesion kinase and inhibits cell migration and integrin-mediated cell spreading and focal adhesion formation. Plays a role as a key modulator of the AKT-mTOR signaling pathway controlling the tempo of the process of newborn neurons integration during adult neurogenesis, including correct neuron positioning, dendritic development and synapse formation. May be a negative regulator of insulin signaling and glucose metabolism in adipose tissue. The nuclear monoubiquitinated form possesses greater apoptotic potential, whereas the cytoplasmic nonubiquitinated form induces less tumor suppressive ability. In motile cells, suppresses the formation of lateral pseudopods and thereby promotes cell polarization and directed movement.	26504226
RB1	RB Transcriptional Corepressor 1	BLCA (IntOGen); GBM (IntOGen)	Key regulator of entry into cell division that acts as a tumor suppressor. Promotes G0-G1 transition when phosphorylated by CDK3/cyclin-C. Acts as a transcription repressor of E2F1 target genes. The underphosphorylated, active form of RB1 interacts with E2F1 and represses its transcription activity, leading to cell cycle arrest. Directly involved in heterochromatin formation by maintaining overall chromatin structure and, in particular, that of constitutive heterochromatin by stabilizing histone methylation. Recruits and targets histone methyltransferases SUV39H1, KMT5B and KMT5C, leading to epigenetic transcriptional repression. Controls histone H4 'Lys-20' trimethylation. Inhibits the intrinsic kinase activity of TAF1. Mediates transcriptional repression by SMARCA4/BRG1 by recruiting a histone deacetylase (HDAC) complex to the c-FOS promoter. In resting neurons, transcription of the c-FOS promoter is inhibited by BRG1-dependent recruitment of a phospho-RB1-HDAC1 repressor complex. Upon calcium influx, RB1 is dephosphorylated by calcineurin, which leads to release of the repressor complex . In case of viral infections, interactions with SV40 large T antigen, HPV E7 protein or adenovirus E1A protein induce the disassembly of RB1-E2F1 complex thereby disrupting RB1's activity.	15084261
RBM5	RNA Binding Motif Protein 5	BRCA (IntOGen)	Component of the spliceosome A complex. Regulates alternative splicing of a number of mRNAs. May modulate splice site pairing after recruitment of the U1 and U2 snRNPs to the 5' and 3' splice sites of the intron. May both positively and negatively regulate apoptosis by regulating the alternative splicing of several genes involved in this process, including FAS and CASP2/caspase-2. In the case of FAS, promotes exclusion of exon 6 thereby producing a soluble form of FAS that inhibits apoptosis. In the case of CASP2/caspase-2, promotes exclusion of exon 9 thereby producing a catalytically active form of CASP2/Caspase-2 that induces apoptosis.	10949932; 12207175; 15192330; 18851835; 18840686
RPL5	Ribosomal Protein L5	GBM (IntOGen)	Required for rRNA maturation and formation of the 60S ribosomal subunits. This protein binds 5S RNA.	19061985
RUNX1	Runt Related Transcription Factor 1	BRCA (IntOGen)	CBF binds to the core site, 5'-PYGPYGGT-3', of a number of enhancers and promoters, including murine leukemia virus, polyomavirus enhancer, T-cell receptor enhancers, LCK, IL-3 and GM-CSF promoters. The alpha subunit binds DNA and appears to have a role in the development of normal hematopoiesis. Isoform AML-1L interferes with the transactivation activity of RUNX1. Acts synergistically with ELF4 to transactivate the IL-3 promoter and with ELF2 to transactivate the mouse BLK promoter. Inhibits KAT6B-dependent transcriptional activation. Controls the anergy and suppressive function of regulatory T-cells (Treg) by associating with FOXP3. Activates the expression of IL2 and IFNG and down-regulates the expression of TNFRSF18, IL2RA and CTLA4, in conventional T-cells.	10207087; 11965546; 14970218; 17431401; 17377532
SETDB1	SET Domain Bifurcated 1	BRCA (IntOGen)	Histone methyltransferase that specifically trimethylates 'Lys-9' of histone H3. H3 'Lys-9' trimethylation represents a specific tag for epigenetic transcriptional repression by recruiting HP1 (CBX1, CBX3 and/or CBX5) proteins to methylated histones. Mainly functions in euchromatin regions, thereby playing a central role in the silencing of euchromatic genes. H3 'Lys-9' trimethylation is coordinated with DNA methylation. Probably forms a complex with MBD1 and ATF7IP that represses transcription and couples DNA methylation and histone 'Lys-9' trimethylation. Its activity is dependent on MBD1 and is heritably maintained through DNA replication by being recruited by CAF-1. SETDB1 is targeted to histone H3 by TRIM28/TIF1B, a factor recruited by KRAB zinc-finger proteins. Probably forms a corepressor complex required for activated KRAS-mediated promoter hypermethylation and transcriptional silencing of tumor suppressor genes (TSGs) or other tumor-related genes in colorectal cancer (CRC) cells. Also required to maintain a transcriptionally repressive state of genes in undifferentiated embryonic stem cells (ESCs). Associates at promoter regions of tumor suppressor genes (TSGs) leading to their gene silencing. The SETDB1-TRIM28-ZNF274 complex may play a role in recruiting ATRX to the 3'-exons of zinc-finger coding genes with atypical chromatin signatures to establish or maintain/protect H3K9me3 at these transcriptionally active regions.	12869583; 14536086; 15327775; 17952062; 24623306; 27029610
SF3B1	Splicing Factor 3b Subunit 1	BRCA (IntOGen); BLCA (IntOGen)	Subunit of the splicing factor SF3B required for 'A' complex assembly formed by the stable binding of U2 snRNP to the branchpoint sequence (BPS) in pre-mRNA. Sequence independent binding of SF3A/SF3B complex upstream of the branch site is essential, it may anchor U2 snRNP to the pre-mRNA. May also be involved in the assembly of the 'E' complex. Belongs also to the minor U12-dependent spliceosome, which is involved in the splicing of rare class of nuclear pre-mRNA intron.	26742993; 25428262
SMC1A	Structural Maintenance Of Chromosomes 1A	BLCA (IntOGen)	Involved in chromosome cohesion during cell cycle and in DNA repair. Central component of cohesin complex. The cohesin complex is required for the cohesion of sister chromatids after DNA replication. The cohesin complex apparently forms a large proteinaceous ring within which sister chromatids can be trapped. At anaphase, the complex is cleaved and dissociates from chromatin, allowing sister chromatids to segregate. The cohesin complex may also play a role in spindle pole assembly during mitosis. Involved in DNA repair via its interaction with BRCA1 and its related phosphorylation by ATM, or via its phosphorylation by ATR. Works as a downstream effector both in the ATM/NBS1 branch and in the ATR/MSH2 branch of S-phase checkpoint.	11877377
TAF1	TATA-Box Binding Protein Associated Factor 1	BRCA (IntOGen)	Largest component and core scaffold of the TFIID basal transcription factor complex. Contains novel N- and C-terminal Ser/Thr kinase domains which can autophosphorylate or transphosphorylate other transcription factors. Phosphorylates TP53 on 'Thr-55' which leads to MDM2-mediated degradation of TP53. Phosphorylates GTF2A1 and GTF2F1 on Ser residues. Possesses DNA-binding activity. Essential for progression of the G1 phase of the cell cycle. Exhibits histone acetyltransferase activity towards histones H3 and H4.	2038334; 8450888; 9660973; 9858607; 11278496; 15053879;
TAOK2	TAO Kinase 2	HNSC (IntOGen)	Serine/threonine-protein kinase involved in different processes such as membrane blebbing and apoptotic bodies formation DNA damage response and MAPK14/p38 MAPK stress-activated MAPK cascade. Phosphorylates itself, MBP, activated MAPK8, MAP2K3, MAP2K6 and tubulins. Activates the MAPK14/p38 MAPK signaling pathway through the specific activation and phosphorylation of the upstream MAP2K3 and MAP2K6 kinases. In response to DNA damage, involved in the G2/M transition DNA damage checkpoint by activating the p38/MAPK14 stress-activated MAPK cascade, probably by mediating phosphorylation of upstream MAP2K3 and MAP2K6 kinases. Isoform 1, but not isoform 2, plays a role in apoptotic morphological changes, including cell contraction, membrane blebbing and apoptotic bodies formation. This function, which requires the activation of MAPK8/JNK and nuclear localization of C-terminally truncated isoform 1, may be linked to the mitochondrial CASP9-associated death pathway. Isoform 1 binds to microtubules and affects their organization and stability independently of its kinase activity. Prevents MAP3K7-mediated activation of CHUK, and thus NF-kappa-B activation, but not that of MAPK8/JNK. May play a role in the osmotic stress-MAPK8 pathway. Isoform 2, but not isoform 1, is required for PCDH8 endocytosis. Following homophilic interactions between PCDH8 extracellular domains, isoform 2 phosphorylates and activates MAPK14/p38 MAPK which in turn phosphorylates isoform 2. This process leads to PCDH8 endocytosis and CDH2 cointernalization. Both isoforms are involved in MAPK14 phosphorylation.	10660600; 13679851; 11279118; 12665513; 17396146;
TBL1XR1	Transducin Beta Like 1 X-Linked Receptor 1	BRCA (IntOGen)	F-box-like protein involved in the recruitment of the ubiquitin/19S proteasome complex to nuclear receptor-regulated transcription units. Plays an essential role in transcription activation mediated by nuclear receptors. Probably acts as integral component of the N-Cor corepressor complex that mediates the recruitment of the 19S proteasome complex, leading to the subsequent proteasomal degradation of N-Cor complex, thereby allowing cofactor exchange, and transcription activation.	14980219
TBX3	T-Box 3	BRCA (CGC & IntOGen)	Transcriptional repressor involved in developmental processes. Probably plays a role in limb pattern formation. Acts as a negative regulator of PML function in cellular senescence.	10468588; 22002537
TGFBR2	Transforming Growth Factor Beta Receptor 2	HNSC (CGC & IntOGen)	Transmembrane serine/threonine kinase forming with the TGF-beta type I serine/threonine kinase receptor, TGFBR1, the non-promiscuous receptor for the TGF-beta cytokines TGFB1, TGFB2 and TGFB3. Transduces the TGFB1, TGFB2 and TGFB3 signal from the cell surface to the cytoplasm and is thus regulating a plethora of physiological and pathological processes including cell cycle arrest in epithelial and hematopoietic cells, control of mesenchymal cell proliferation and differentiation, wound healing, extracellular matrix production, immunosuppression and carcinogenesis. The formation of the receptor complex composed of 2 TGFBR1 and 2 TGFBR2 molecules symmetrically bound to the cytokine dimer results in the phosphorylation and the activation of TGFRB1 by the constitutively active TGFBR2. Activated TGFBR1 phosphorylates SMAD2 which dissociates from the receptor and interacts with SMAD4. The SMAD2-SMAD4 complex is subsequently translocated to the nucleus where it modulates the transcription of the TGF-beta-regulated genes. This constitutes the canonical SMAD-dependent TGF-beta signaling cascade. Also involved in non-canonical, SMAD-independent TGF-beta signaling pathways.	7774578
TP53	Tumor Protein P53	BLCA (IntOGen)	Acts as a tumor suppressor in many tumor types; induces growth arrest or apoptosis depending on the physiological circumstances and cell type. Involved in cell cycle regulation as a trans-activator that acts to negatively regulate cell division by controlling a set of genes required for this process. One of the activated genes is an inhibitor of cyclin-dependent kinases. Apoptosis induction seems to be mediated either by stimulation of BAX and FAS antigen expression, or by repression of Bcl-2 expression. In cooperation with mitochondrial PPIF is involved in activating oxidative stress-induced necrosis; the function is largely independent of transcription. Induces the transcription of long intergenic non-coding RNA p21 (lincRNA-p21) and lincRNA-Mkln1. LincRNA-p21 participates in TP53-dependent transcriptional repression leading to apoptosis and seem to have to effect on cell-cycle regulation. Implicated in Notch signaling cross-over. Prevents CDK7 kinase activity when associated to CAK complex in response to DNA damage, thus stopping cell cycle progression. Isoform 2 enhances the transactivation activity of isoform 1 from some but not all TP53-inducible promoters. Isoform 4 suppresses transactivation activity and impairs growth suppression mediated by isoform 1. Isoform 7 inhibits isoform 1-mediated apoptosis. Regulates the circadian clock by repressing CLOCK-ARNTL/BMAL1-mediated transcriptional activation of PER2.	9840937; 11025664; 12810724; 15186775; 19556538; 24051492
TSC1	Tuberous Sclerosis 1	BLCA (CGC & IntOGen)	In complex with TSC2, inhibits the nutrient-mediated or growth factor-stimulated phosphorylation of S6K1 and EIF4EBP1 by negatively regulating mTORC1 signaling. Seems not to be required for TSC2 GAP activity towards RHEB. Implicated as a tumor suppressor. Involved in microtubule-mediated protein transport, but this seems to be due to unregulated mTOR signaling.	12271141; 15340059
WHSC1	Wolf-Hirschhorn syndrome candidate 1	HNSC (IntOGen)	Histone methyltransferase with histone H3 'Lys-27' (H3K27me) methyltransferase activity. Isoform 2 may act as a transcription regulator that binds DNA and suppresses IL5 transcription through HDAC recruitment.	11152655; 16115125; 18172012
ZFP36L1	ZFP36 Ring Finger Protein Like 1	BRCA (IntOGen)	Zinc-finger RNA-binding protein that destabilizes several cytoplasmic AU-rich element (ARE)-containing mRNA transcripts by promoting their poly(A) tail removal or deadenylation, and hence provide a mechanism for attenuating protein synthesis. Acts as a 3'-untranslated region (UTR) ARE mRNA-binding adapter protein to communicate signaling events to the mRNA decay machinery. Functions by recruiting the CCR4-NOT deadenylase complex and components of the cytoplasmic RNA decay machinery to the bound ARE-containing mRNAs, and hence promotes ARE-mediated mRNA deadenylation and decay processes. Induces also the degradation of ARE-containing mRNAs even in absence of poly(A) tail . Binds to 3'-UTR ARE of numerous mRNAs. Positively regulates early adipogenesis by promoting ARE-mediated mRNA decay of immediate early genes (IEGs) . Promotes ARE-mediated mRNA decay of mineralocorticoid receptor NR3C2 mRNA in response to hypertonic stress. Negatively regulates hematopoietic/erythroid cell differentiation by promoting ARE-mediated mRNA decay of the transcription factor STAT5B mRNA. Positively regulates monocyte/macrophage cell differentiation by promoting ARE-mediated mRNA decay of the cyclin-dependent kinase CDK6 mRNA. Promotes degradation of ARE-containing pluripotency-associated mRNAs in embryonic stem cells (ESCs), such as NANOG, through a fibroblast growth factor (FGF)-induced MAPK-dependent signaling pathway, and hence attenuates ESC self-renewal and positively regulates mesendoderm differentiation . May play a role in mediating pro-apoptotic effects in malignant B-cells by promoting ARE-mediated mRNA decay of BCL2 mRNA. In association with ZFP36L2 maintains quiescence on developing B lymphocytes by promoting ARE-mediated decay of several mRNAs encoding cell cycle regulators that help B cells progress through the cell cycle, and hence ensuring accurate variable-diversity-joining (VDJ) recombination and functional immune cell formation . Together with ZFP36L2 is also necessary for thymocyte development and prevention of T-cell acute lymphoblastic leukemia (T-ALL) transformation by promoting ARE-mediated mRNA decay of the oncogenic transcription factor NOTCH1 mRNA . Participates in the delivery of target ARE-mRNAs to processing bodies (PBs). In addition to its cytosolic mRNA-decay function, plays a role in the regulation of nuclear mRNA 3'-end processing; modulates mRNA 3'-end maturation efficiency of the DLL4 mRNA through binding with an ARE embedded in a weak noncanonical polyadenylation (poly(A)) signal in endothelial cells. Also involved in the regulation of stress granule (SG) and P-body (PB) formation and fusion. Plays a role in vasculogenesis and endocardial development . Plays a role in the regulation of keratinocyte proliferation, differentiation and apoptosis. Plays a role in myoblast cell differentiation	12198173; 15967811; 17030608; 19179481; 25106868; 27182009

Supplementary Table S4. AUC scores of PR-curves of the detection results for IntOGen genes in BRCA, BLCA, GBM, HNSC and LAML.

Database	IntOGen				
Method	BRCA	BLCA	GBM	HNSC	LAML
mCGfinder	13.0%	10.5%	13.2%	9.6%	66.7%
HotNet2	2.4%	1.8%	1.1%	1.9%	9.4%
ReMIC	5.3%	3.2%	3.0%	3.8%	0.6%
Random	1.5%	1.3%	0.6%	1.3%	0.2%
Database	Union(CGC, IntOGen)				
Method	BRCA	BLCA	GBM	HNSC	LAML
mCGfinder	13.0%	10.9%	12.8%	9.9%	26.5%
HotNet2	2.5%	1.9%	1.2%	1.9%	6.7%
ReMIC	5.4%	3.3%	3.2%	4.1%	1.6%
Random	1.5%	1.3%	0.7%	1.4%	0.7%

Supplementary Table S5. The detailed information of TCGA somatic mutation datasets of RBCA, BLCA, GBM, HNSC and LAML respectively. The datasets are downloaded from the UCSC Cancer Genomics Browser [11]: https://genome-cancer.soe.ucsc.edu/proj/site/hgHeatmap/

Title	TCGA bladder urothelial carcinoma (BLCA) gene-level nonsilent somatic mutation (broad automated)
Dataset	BLCA gene-level mutation (broad automated)
Dataset ID	TCGA_BLCA_mutation_broad_gene
Domain	TCGA
Origin	Bladder
Disease	bladder urothelial carcinoma
Sample Type	tumor
Data Type	somatic mutation
Clinical Cohort	TCGA Bladder Cancer
N	238
Version	2015-02-24
Title	TCGA breast invasive carcinoma (BRCA) gene-level nonsilent somatic mutation (wustl)
Dataset	BRCA gene-level mutation (wustl)
Dataset ID	TCGA_BRCA_mutation_wustl_gene
Domain	TCGA
Origin	Breast
Disease	breast invasive carcinoma
Sample Type	tumor
Data Type	somatic mutation
Clinical Cohort	TCGA Breast Cancer
N	776
Version	2015-02-24
Title	TCGA glioblastoma multiforme (GBM) gene-level nonsilent somatic mutation (broad)
Dataset	GBM gene-level mutation (broad)
Dataset ID	TCGA_GBM_mutation_broad_gene
Domain	TCGA
Origin	Brain
Disease	glioblastoma multiforme
Sample Type	tumor
Data Type	somatic mutation
Clinical Cohort	TCGA Glioblastoma
N	291
Version	2015-02-24

continued on next page

continued from previous page

Title	TCGA head & neck squamous cell carcinoma (HNSC) gene-level nonsilent somatic mutation (broad automated)
Dataset	HNSC gene-level mutation (broad automated)
Dataset ID	TCGA_HNSC_mutation_broad_gene
Domain	TCGA
Origin	Head and Neck region
Disease	head & neck squamous cell carcinoma
Sample Type	tumor
Data Type	somatic mutation
Clinical Cohort	TCGA Head and Neck Cancer
N	509
Version	2015-02-24
Title	TCGA acute myeloid leukemia (LAML) gene-level nonsilent somatic mutation (wustl)
Dataset	LAML gene-level mutation (wustl)
Dataset ID	CGA_LAML_mutation_wustl_gene
Domain	TCGA
Origin	White blood cell
Disease	acute myeloid leukemia
Sample Type	tumor
Data Type	somatic mutation
Clinical Cohort	TCGA Acute Myeloid Leukemia
N	197
Version	2015-02-24
